# *In vivo* and *in silico* analysis of PCNA ubiquitylation in the activation of the Post Replication Repair pathway in *S. cerevisiae*

**DOI:** 10.1186/1752-0509-7-24

**Published:** 2013-03-20

**Authors:** Flavio Amara, Riccardo Colombo, Paolo Cazzaniga, Dario Pescini, Attila Csikász-Nagy, Marco Muzi Falconi, Daniela Besozzi, Paolo Plevani

**Affiliations:** 1 Dipartimento di Bioscienze, Università degli Studi di Milano, Milano, Italy; 2 Dipartimento di Informatica, Sistemistica e Comunicazione, Università degli Studi di Milano-Bicocca, Milano, Italy; 3 Dipartimento di Scienze Umane e Sociali, Università degli Studi di Bergamo, Bergamo, Italy; 4 Dipartimento di Statistica e Metodi Quantitativi, Università degli Studi di Milano-Bicocca, Milano, Italy; 5, The Microsoft Research - Università degli Studi di Trento, Centre for Computational and Systems Biology, Rovereto (Trento), Italy; 6 Dipartimento di Informatica, Università degli Studi di Milano, Milano, Italy

**Keywords:** DNA damage, Post replication repair, PCNA, Ubiquitylation, Budding yeast, Mechanistic modeling, Stochastic simulation, Paramater sweep analysis

## Abstract

**Background:**

The genome of living organisms is constantly exposed to several damaging agents that induce different types of DNA lesions, leading to cellular malfunctioning and onset of many diseases. To maintain genome stability, cells developed various repair and tolerance systems to counteract the effects of DNA damage. Here we focus on Post Replication Repair (PRR), the pathway involved in the bypass of DNA lesions induced by sunlight exposure and UV radiation. PRR acts through two different mechanisms, activated by mono- and poly-ubiquitylation of the DNA sliding clamp, called Proliferating Cell Nuclear Antigen (PCNA).

**Results:**

We developed a novel protocol to measure the time-course ratios between mono-, di- and tri-ubiquitylated PCNA isoforms on a single western blot, which were used as the wet readout for PRR events in wild type and mutant *S. cerevisiae* cells exposed to acute UV radiation doses. Stochastic simulations of PCNA ubiquitylation dynamics, performed by exploiting a novel mechanistic model of PRR, well fitted the experimental data at low UV doses, but evidenced divergent behaviors at high UV doses, thus driving the design of further experiments to verify new hypothesis on the functioning of PRR. The model predicted the existence of a UV dose threshold for the proper functioning of the PRR model, and highlighted an overlapping effect of Nucleotide Excision Repair (the pathway effectively responsible to clean the genome from UV lesions) on the dynamics of PCNA ubiquitylation in different phases of the cell cycle. In addition, we showed that ubiquitin concentration can affect the rate of PCNA ubiquitylation in PRR, offering a possible explanation to the DNA damage sensitivity of yeast strains lacking deubiquitylating enzymes.

**Conclusions:**

We exploited an *in vivo* and *in silico* combinational approach to analyze for the first time in a Systems Biology context the events of PCNA ubiquitylation occurring in PRR in budding yeast cells. Our findings highlighted an intricate functional crosstalk between PRR and other events controlling genome stability, and evidenced that PRR is more complicated and still far less characterized than previously thought.

## Background

The genome of living organisms is constantly exposed to several exogenous and endogenous damaging agents – environmental chemicals, ultraviolet (UV) light, ionizing radiation, reactive oxygen species – that can result in DNA lesions potentially leading to cellular malfunctioning, aging and the onset of several diseases, including cancer and neurodegeneration [1,2]. Since the maintenance of genome stability is a pivotal task for cell survival and division, cells have developed various repair and tolerance systems to counteract the effects of DNA damage, which altogether rely on the crosstalk between the biochemical processes involved in DNA metabolism and in cell cycle progression. According to the type of DNA lesion and the cell cycle stage at which the damage occurs, cells exploit the activation of specific DNA-damage tolerance (DDT) pathways. In this work, we aim to investigate the functioning of Post Replication Repair (PRR), a DDT pathway involved in the recognition of the most abundant mutagenic and cytotoxic DNA lesions induced by sunlight exposure and UV radiation [3-6].

The cellular pathway that is effectively responsible to clean the genome from these UV adducts is Nucleotide Excision Repair (NER). Briefly, NER excides short DNA patches (∼ 30 nucleotides long) containing UV lesions and promotes the filling of the generated single-stranded DNA (ssDNA) gap [7,8]. It is generally assumed that NER acts in the G1 and G2 phases of the cell cycle, as the NER excision activity can result dangerous during S phase because it might generate breaks and gaps near the incoming replication forks [9]. On the other hand, during the S phase of the cell cycle the DNA replication machinery stalls in front of a UV-induced lesion, because of the inability of the replicative DNA polymerases to incorporate nucleotides opposite a UV adduct. To avoid prolonged stalling and the possible collapse of the replication fork, eukaryotic cells activate the PRR pathway, whose role is not to repair but instead to *bypass* the UV-induced DNA lesions, allowing the replisome to complete genome replication over the damaged template. This way, cells can complete S phase and postpone DNA repair at the G2/M transition.

Notwithstanding the relevance of genome integrity and the ever increasing body of data that is continuously produced in this field, a global view of the crosstalk between the numerous DNA repair pathways is still lacking. Recently, a number of studies based on wet experiments, as well as computational modeling and bioinformatics tools, started to investigate these mutual relationships, in order to understand how regulative mechanisms and proteins modifications occurring in each pathway are able to influence the other pathways to coordinate either the detection, the repair or the bypass of the different DNA lesions, in a finely orchestrated manner with cell cycle progression and cellular metabolism [10-16].

In addition, a number of mathematical models were recently defined to analyze in details specific processes that govern the machinery of DNA damage and repair in different organisms. For instance, some works investigated the dynamics of double-strand breaks (DSBs) formation in bacteria, and analyzed the relation between bacterial death rate and the concentration of endogenous damaging agents [17], or tried to explain the UV-induced SOS response in *E. coli* incorporating mutagenesis by error-prone DNA polymerases [18]. In eukaryotes, several models were proposed to analyze different regulatory mechanisms, such as the detection of DSBs depending on ATM (ataxia telangiectasia mutated) autophosphorylation [19], or the imbalance between DNA damage and repair processes in the formation of DNA adducts due to oxidative estrogen metabolism [20]. Most of these models focused on NER and Base Excision Repair (BER) pathways in human and mammalian cells (see, e.g., [21-24]), though no mathematical model was developed up to now to elucidate the mechanisms governing the PRR pathway.

Experimental works concerning this complicated and not well characterized pathway determined that the bypass of UV adducts promoted by PRR involves two different sub-pathways: the first may be error-prone and is related to Translesion DNA Synthesis (TLS), while the second is error-free and acts through Template Switching (TS) processes. The key event driving the activation of these sub-pathways is a post-translational modification of the sliding clamp Proliferating Cell Nuclear Antigen (PCNA), a protein acting as scaffold for the binding of replicative DNA polymerases and several other proteins involved in DNA replication, repair and cell cycle regulation [3,25,26]. In particular, in *Saccharomyces cerevisiae* it is known that the mono-ubiquitylation of PCNA drives the PRR pathway to TLS, while PCNA poly-ubiquitylation directs PRR to the error-free sub-pathway. A major issue in the study of PRR is to understand how the dynamics of PCNA ubiquitylation might influence the choice between TLS and TS, or whether there exists a damage-related threshold able to regulate the crosstalk between these sub-pathways.

In this paper, we focus on the analysis of the events of PCNA ubiquitylation occurring in PRR by exploiting a bottom-up Systems Biology approach, based on data-driven modeling and model-driven experiments. This analysis was carried out through an integrated and cyclic workflow consisting in laboratory experiments based on genetic and molecular biology protocols on the one side, and mathematical modeling and computational analysis on the other side. In particular, experimental measurements of the ratio between mono- and poly-ubiquitylated PCNA were used as the wet readout of the cellular response to acute UV irradiation, carried out through a systematic *in vivo* characterization of the dynamics of PCNA ubiquitylation after UV irradiation of *S. cerevisiae* cells. For this purpose, we developed a specific experimental protocol that allows the detection of mono- and poly-ubiquitylated PCNA isoforms on a single western blot, differently from other previously devised methods which could only allow the measurement of the amount of di- to N-ubiquitylated PCNA isoforms – albeit not the mono-ubiquitylated one – on the same film (see, e.g., [27]). These laboratory measurements were systematically compared with the outcome of stochastic simulations, performed by exploiting a novel mechanistic model of PRR that describes in details the molecular interactions involved in the mono- and poly-ubiquitylation steps of PCNA, and which takes into account the estimated number of induced DNA lesions at different UV doses.

In this context, the choice of a stochastic computational framework was motivated by several aspects related to the pathway under investigation. First of all, the molecular amounts of most species involved in PRR are low, and we also evidenced quite large noise in the experimental measurements; therefore, a stochastic approach was more suitable to capture the possible noise effects in the emergent dynamics of PCNA ubiquitylation. Secondly, the mechanistic approach based on the stochastic formulation of chemical kinetics [28] that we exploited to define the mathematical model of PRR, allows to give a detailed description of the molecular interactions occurring in PRR events, and also to test different interaction topologies while minimizing the model revision from time to time.

In addition, the definition of the mathematical model benefited from a preliminary bioinformatic process based on three-dimensional protein structure modeling, to confirm the actual spatio-temporal cascade of PRR interactions, and on a successive computational phase based on parameter sweep analysis and sensitivity analysis, to test the reliability of the chosen model parameterization.

The integration of *in vivo* and *in silico* data allowed us to make predictions on the functioning of PRR in living cells and to drive the design of further laboratory experiments, aimed at improving the knowledge of this pathway. In particular, our results suggest the existence of a UV dose threshold for the proper functioning of our PRR model in response to UV-induced damages, corroborated by a fine concordance of the balance of mono- and poly-ubiquitylated PCNA isoforms between wet measurements and simulation outcomes at UV doses below 30 J/m^2^. Above this threshold, we obtained an unexpected discrepancy between *in vivo* and *in silico* data, which induced us to postulate an overlapping effect of NER on the dynamics of PCNA ubiquitylation (altering the actual response of PRR in irradiated cells), and the relevance of NER not only in G1 and G2 phases but also during the S phase of the cell cycle, in agreement with some previous observations [29]. In addition, our results showed that the concentration of free ubiquitin in the nuclear compartment can affect the rate of PCNA ubiquitylation, offering a possible explanation to the DNA damage sensitivity of yeast strains lacking deubiquitylating enzymes [30].

After providing a detailed description of the biochemical processes involved in the PRR pathway in budding yeast cells, we present the experimental procedures and the computational methods exploited in this Systems Biology work. Then, we show the computational results related to the definition of the mathematical model and discuss the biological insights concerning PRR, which were achieved thanks to the combined crosstalk of *in silico* simulations and laboratory experiments. Finally, we conclude the paper with some final remarks and open questions for future research.

### Post Replication Repair in *S. cerevisiae*

PRR is the most complicated and least characterized DDT pathway [3] involved in the bypass of the most abundant mutagenic and cytotoxic DNA lesions induced by sunlight exposure and UV radiation, namely, pyrimidine cyclobutane dimers (CPDs) and 6-4 photoproducts (6-4 PPs) [4-6]. The key event process taking place in PRR for the bypass of these UV lesions is the ubiquitylation at lysine 164 (K164) of the sliding clamp named Proliferating Cell Nuclear Antigen (PCNA) [3,25,26]. PCNA is a ring-shaped homotrimeric protein, which encircles and slides along double-stranded DNA molecules localizing and tethering a plethora of other proteins (such as polymerases) to DNA. PCNA ubiquitylation in response to UV-induced DNA damage does not mediate proteasomal-induced degradation: rather, K164 PCNA ubiquitylation signals the presence of a non-replicable UV lesion in the genome [31,32].

The ubiquitylation process of PCNA requires three consecutive steps: (1) ubiquitin activation, (2) ubiquitin trans-thio-esterification, and (3) PCNA ubiquitin conjugation. While the first two steps are common to other cellular pathways, the third step is carried out by proteins that are specific of PRR. In *S. cerevisiae*, the step of ubiquitin activation is mediated by Uba1, which is the unique ubiquitin activating enzyme (also known as E1) in budding yeast. This process, requiring ATP consumption, is fully conserved in eukaryotes and it requires at least 20 biochemical reactions on the whole [33]. Once activated, the ubiquitin moves from the E1 enzyme to the ubiquitin conjugating enzyme (also known as E2) through a trans-thio-esterification reaction. In *S. cerevisiae*, the PRR pathway includes two different E2 enzymes which are able to receive an activated ubiquitin from Uba1: Rad6 and Ubc13. The latter enzyme, Ubc13, works in a complex with an ubiquitin conjugating enzyme variant (UEV), called Mms2 [34,35]. The key event in the activation of PRR actually consists in the capability of transferring the ubiquitin from an E2 enzyme to PCNA, through the action of an ubiquitin ligase enzyme (also known as E3). In *S. cerevisiae* cells there exist two different E3 enzymes, Rad18 and Rad5, which drive the ubiquitin transfer to PCNA from the E2 Rad6 and Ubc13-Mms2, respectively. Each E3 enzyme has its own function and specificity within the PRR pathway, and the kind of ubiquitylation they carry out on PCNA drives the next steps of the DNA damage bypass process in different ways.

**Figure 1:**
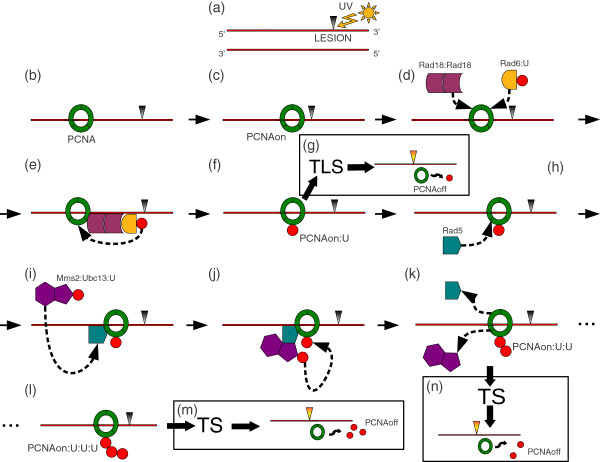
**Graphical representation of the PRR pathway.** The diagram shows the main steps of the PRR pathway concerning the covalent modification of PCNA (mono- and poly-ubiquitylation) in response to UV-induced damage. For simplicity, in the diagram the replication fork is represented just by its PCNA component. UV radiation can induce lesions on DNA (represented as a gray triangle in step (**a**)), which cause the stall of the replication fork (denoted by *PCNA*_on_)(steps (**b**, **c**), corresponding to reaction 1 in Table 3). Afterwards, PCNA is mono-ubiquitylated (*PCNA*_on_:*U*) by the combined activity of E2 Rad6 and E3 Rad18 (steps (**d**, **e**, **f**), corresponding to reactions 2 to 10 in Table 3). At this stage, mono-ubiquitylated PCNA can activate the Translesion DNA Synthesis sub-pathway (TLS), leading to lesion bypass (represented as an orange triangle in step (**g**), corresponding to reaction 23 in Table 3), and eventually to the ubiquitylation signal switch-off (*PCNA*_off_). On the other hand, mono-ubiquitylated PCNA can undergo further ubiquitylation events through the combined action of E2 Ubc13-Mms2 and E3 Rad5 (steps (**h**, **i**, **j**), corresponding to reactions 11 to 16 in Table 3), adding a single ubiquitin moiety per step (step (**k**), corresponding to reaction 17 in Table 3, and step (**l**), corresponding to reaction 22 in Table 3). Di- and tri-ubiquitylated PCNA isoforms are denoted by *PCNA*_on_:*U*:*U* (**k**) and *PCNA*_on_:*U*:*U*:*U* (**l**), respectively. The steps for PCNA tri-ubiquitylation are not formally represented (dotted steps between (**k**) and (**l**), corresponding to reactions 18 to 20 in Table 3), being the process analogous to steps (**h**, **i**, **j**, **k**) for di-ubiquitylation. Poly-ubiquitylated PCNA promotes the lesion bypass (represented as an orange triangle in step (**m**), corresponding to reaction 25 in Table 3, and in step (**n**), corresponding to reaction 24 in Table 3) through the Template Switching sub-pathway (TS) which, eventually, leads to the ubiquitylation signal switch-off.

On the whole, the ubiquitylation process within the PRR pathway involves five actors (see Figure 1): two E2 enzymes (Rad6 and Ubc13-Mms2), two E3 enzymes (Rad18 and Rad5) and one target (PCNA) [25]. The way the E2 and E3 enzymes work together to induce the covalent modification of PCNA was recently characterized *in vitro* by using purified *S. cerevisiae* proteins [36], suggesting that the most probable mechanism of PCNA ubiquitin conjugation after UV-induced DNA damage consists in the following step-wise process. After stalling of the replication machinery, the formation of RPA protein-coated ssDNA at level of the UV lesion leads to the recruitment of the Rad18-Rad6 complex and the subsequent mono-ubiquitylation of PCNA at K164 [37]. This mono-ubiquitylation can be further extended by Rad5 and Ubc13-Mms2, which carry another ubiquitin moiety. Then, the Rad5-Ubc13-Mms2 complex can further bind to di-ubiquitylated PCNA and, in a step-wise process, it leads to tri-ubiquitylation and, rarely, to tetra-ubiquitylation of PCNA [38]. This limited PCNA poly-ubiquitylation is obtained through the formation of K63-linked ubiquitin chains, and this linkage specificity is the major signal for UV lesion bypass.

It is effectively the balance between the mono- and poly-ubiquitylated modification states of PCNA that is able to influence the distinct modes of UV lesion bypass: mono-ubiquitylation drives the PRR towards Translesion DNA Synthesis (TLS), while K63-linked poly-ubiquitylation drives it to Template Switching (TS). Regarding TLS, PCNA mono-ubiquitylation enhances the binding affinity of particular DNA polymerases, called TLS polymerases (Pol- *η*, Rev1 and Pol- *ζ* in budding yeast), which are able to substitute the stalled replicative polymerases in a process called “polymerase switch” [39]. TLS polymerases are able to host the UV lesion within their active site and to incorporate a nucleotide in front of the lesion, while the replicative polymerases Pol- *δ* and Pol- *ε* are unable to bypass the DNA damage. Depending on which TLS polymerase binds PCNA, two different kinds of UV lesion bypass are possible. In fact, TLS can be further divided into two sub-branches: the “error-free TLS”, taking place when Pol- *η* incorporates the right nucleotide opposite a CPD, and the “error-prone TLS”, occurring when Pol- *η* incorporates a wrong nucleotide opposite a 6-4 PP and then stalls. In this case, Pol- *η* is replaced by Pol- *ζ* which, in cooperation with Rev1, is able to carry on the replication, though generating mutations on the genome [40,41]. Therefore, PCNA mono-ubiquitylation and TLS are potentially mutagenic and may cause genome instability. Conversely, the TS sub-pathway allows to bypass UV lesions in an error-free way, because it exploits the invasion of the undamaged DNA sister filament to overcome replication fork stalling, without the intervention of TLS polymerases (a process also known as recombination-based UV lesion bypass). This branch of PRR is enhanced by PCNA K63-linked poly-ubiquitylation, but the molecular details of the whole process are largely unknown.

A relevant issue in the investigation of PRR is related to the regulation of the dynamics of PCNA ubiquitylation in response to UV-induced damages, since the balance between its mono- and poly-ubiquitylated isoforms is supposed to control the crosstalk between the TLS and TS sub-pathways. Therefore, we propose hereby a detailed mechanistic model of the enzymatic processes involved in PCNA ubiquitylation related to the PRR pathway, in order to monitor the formation of mono- versus poly- ubiquitylated PCNA isoforms after UV irradiation, and to better understand the cellular response to UV-induced DNA damages.

## Methods

### Experimental procedures

#### *S. cerevisiae* strains, media and growing conditions

All *S. cerevisiae* strains used in this work are isogenic to DF5 genetic background (*his3-δ200 leu2-3,112 lys2-801 trp1-1 ura3-52*) and deletion mutants were constructed by one-step PCR strategy [42] and confirmed by PCR [43]. All strains are carrying the ^HIS^*POL30* allele, expressing HIS tagged PCNA protein. All strains were grown in liquid YPD following standard genetic procedures [43]. The complete list of strains used in this work is given in Table 1.

**Table 1:** Yeast strains used in this study

**Strain name**	**Genotype**	**Reference**
WT	(DF5) *pol30δ*::URA3 LEU2::YIplac128-His_6_-*POL30*	[38]
*rad14δ*	(DF5) *pol30δ*::URA3 LEU2::YIplac128-His_6_-*POL30rad14δ*::KanMX6	This study
*doa4δ*	(DF5) *pol30δ*::URA3 LEU2::YIplac128-His_6_-*POL30doa4δ*::KanMX6	This study
*ubp10δ*	(DF5) *pol30δ*::URA3 LEU2::YIplac128-His_6_-*POL30ubp10δ*::KanMX6	This study
*ubp15δ*	(DF5) *pol30δ*::URA3 LEU2::YIplac128-His_6_-*POL30ubp15δ*::KanMX6	This study
*ubp10δubp15δ*	(DF5) *pol30δ*::URA3 LEU2::YIplac128-His_6_-*POL30ubp15δ*::KanMX6 *ubp10δ*::TRP1	This study

#### UV irradiation

Yeast cells were grown to a concentration of ∼ 10^7^ cells/ml, plated on YPD agar and irradiated with different doses (5 to 75 J/m^2^) of UV-C light (254 nm). Immediately after irradiation, cells were resuspended in liquid medium and samples collected at various time points – taken between 0 h and 5 h after irradiation – were analyzed. In order to avoid variations due to newly synthesized proteins which may affect experimental reproducibility and alter the comparison with the computational modeling, we added 10 *μ*g/ml of cycloheximide (CHX), a protein synthesis inhibitor, after UV irradiation of logarithmically growing cells. This CHX dose inhibits protein synthesis to ∼ 90% within 15 min [44] and stops cell cycle progression, as shown in histogram and density plots reported in Additional file 1.

#### Fluorescence activated cell sorter (FACS) analysis

Cell cycle progression was monitored by measuring DNA content by FACS analysis, as previously described [45]. By this analysis we established the percentage of G1, S and G2 cells in a cell population.

#### *In vivo* detection of ubiquitylated PCNA and signal quantification

Ubiquitylated PCNA represents a small percentage of total PCNA in the cells, and quantitative detection of the modified PCNA isoforms requires their enrichment. This was achieved by using yeast strains carrying the ^HIS^*POL30* allele and a well established pull-down protocol [46]. Before pull-down of ubiquitylated PCNA, cell lisates were normalized to the sample containing the lower amount of proteins determined with the BioRad Assay. Proteins were separated by PAGE on 10% SDS-urea [47] and then transferred onto a nitrocellulose membrane at 4°C. After transfer, nitrocellulose membranes were treated at 120°C for 30 min. Ubiquitylated PCNA was detected with polyclonal anti-Ub antibody, and unmodified PCNA was detected with a monoclonal anti-HIS antibody (Cell Signaling). The signal corresponding to ubiquitylated PCNA was quantified by ImageJ software [48]. The PCNA ubiquitylation signal was normalized to the unmodified PCNA signal and analyzed as the ratio between mono-, di- and tri-ubiquitylated PCNA, as described in Additional file 2.

### Model parameterization

In order to gain insights into the functioning of a biological system, it is fundamental to identify the system structure (i.e., its main components and their respective interactions), as well as the set of parameters involved, which are needed to perform simulations and to conduct a quantitative analysis of the system response under different conditions. Here, we present a mechanistic model of the PRR pathway, defined according to the stochastic formulation of chemical kinetics [28], which requires to specify the set of molecular species occurring in the pathway and their respective interactions, formally described as a set of biochemical reactions.

The parameterization of this model is given by the values of the stochastic constants associated to the reactions, the molecular amounts of the species initially present in the system, and the number of lesions per genome corresponding to any given UV irradiation dose. We describe hereafter the methods used to determine and calibrate the values of the parameters of the PRR model, and to analyze the PCNA ubiquitylation dynamics in response to UV-induced damages.

#### Molecular species amounts

*In vivo* data related to PRR protein concentrations were retrieved from [49], the Saccharomyces Genome Database (SGD) [50,51], and VonDerHaar curated database [52], which represent the primary sources for the identification of the amount of proteins within yeast cells.

Since the model describes a process taking place inside the nucleus, the molecular amounts of the PRR proteins that are localized both in the nucleus and in the cytoplasm (Rad6, Ubc13, Mms2 and ubiquitin) had to be scaled to the nuclear portion volume. To this aim, since we could not retrieve any additional information about the precise cellular localization (related to, e.g., concentration gradients or buffering mechanisms) of all the proteins involved in PRR, we assumed a uniform distribution within the cell. Then, we calculated the nuclear amount of these proteins as a fraction of their whole cellular amount (as reported in the protein databases), proportional to the nuclear volume of the cell. In *S. cerevisiae*, the nuclear volume corresponds to 7% of the total cell volume for exponential growing cells, as derived through microscopy techniques [53].

The derived molecular amounts (expressed as number of molecules per cell) of the proteins occurring in the initial state of the PRR model are reported in Table 2.

**Table 2:** Molecular amounts of initial species in the PRR model

**Protein**	**Total amount**	**Reference**	**Nuclear amount**
Rad5	1520	[50]	1520
Rad6	2770	[50]	194
Rad18	206	[50]	206
Ubc13*	8970	[50]	628
Mms2*	2760	[50]	193
PCNA	22440 (7480 trimers)	[52]	7480 trimers
Ubiquitin	124260	[50,52]	8698

The molecular amounts of the proteins initially occurring in the PRR model are here expressed as number of molecules per cell. Data in the second column were collected from log phase growing yeast cells, retrieved from the reference database reported in the third column. The amount of ubiquitin is calculated as the sum of molecular amounts deriving from the four genes coding for ubiquitin in yeast cells (namely, UBI1, UBI2, UBI3, UBI4). The fourth column reports the derived nuclear amounts of the proteins that are localized both in the nucleus and in the cytoplasm, scaled according to the 7% fraction of nuclear volume with respect to the total yeast cell volume [53]. This column specifies, in particular, the molecular amounts of proteins occurring in the initial state of the system. The proteins marked with the asterisk (Ubc13, Mms2) occur in the model as complex (Ubc13 : Mms2), whose initial amount is fixed at the minimum value between the amounts of the two proteins (namely, 193 molecules/cell).

#### Reaction constants

In the context of computational modeling, the general lack of experimental measurements of *in vivo* kinetics usually challenges the definition of a homogeneous set of values for reaction constants. As a matter of fact, the available literature on PRR is almost characterized by qualitative descriptions of the pathway; the only kinetic constant that we could assess from literature corresponds to the process of poly-ubiquityl chain extension on mono-ubiquitylated PCNA, showing a *K*_cat_=(3.0±0.04) min−1 [54]. This value was transformed into the stochastic constant of the corresponding reaction in the model (Table 3, reaction 16), according to the correspondence between deterministic rate constants and stochastic constants given in [28].

**Table 3:** Mechanistic model of the PRR pathway in budding yeast

**Reaction**	**Reagents**	**Products**	**Constant*c***_***i***_**[sec**^**−1**^**]**	**Reference**	**Step in Figure**1
1	*PCNA*+*L*	*PCNA*_on_	1.5 ×10^−8^	This study	**c**
2	*Rad*18+*Rad*18	*Rad*18:*Rad*18	1 ×10^−2^	[55,56]	
3	*Rad*18:*Rad*18	*Rad*18+*Rad*18	1 ×10^3^	[55,56]	
4	*Rad*6+*U*	*Rad*6:*U*	2.5 ×10^−7^	[57]	
5	*PCNA*_on_+*Rad*18:*Rad*18	*Rad*18:*Rad*18:*PCNA*_on_	1 ×10^5^	[25,36]	**d**
6	*Rad*18:*Rad*18:*PCNA*_on_	*PCNA*_on_+*Rad*18:*Rad*18	1 ×10^3^	[25,36]	
7	*Rad*6:*U*+*Rad*18:*Rad*18:*PCNA*_on_	*Rad*18:*Rad*18:*PCNA*_on_:*Rad*6:*U*	3.51 ×10^−2^	[25,36,58-61], this study	
8	*Rad*18:*Rad*18:*PCNA*_on_:*Rad*6:*U*	*Rad*6:*U*+*Rad*18:*Rad*18:*PCNA*_on_	1 ×10^−2^	[25,36,58-61], this study	
9	*Rad*18:*Rad*18:*PCNA*_on_:*Rad*6:*U*	*Rad*6+*Rad*18:*Rad*18:*PCNA*_on_:*U*	1 ×10^−2^	[25,36,60]	**e**
10	*Rad*18:*Rad*18:*PCNA*_on_:*U*	*Rad*18:*Rad*18+*PCNA*_on_:*U*	1	[25,36,60]	**e**, **f**
11	*Ubc*13:*Mms*2+*U*	*Ubc*13:*U*:*Mms*2	1 ×10^5^	[35,62,63], this study	**i**
12	*PCNA*_on_:*U*+*Rad*5	*Rad*5:*PCNA*_on_:*U*	5 ×10^−6^	[36]	**h**
13	*Rad*5:*PCNA*_on_:*U*	*PCNA*_on_:*U*+*Rad*5	5 ×10^−3^	[36]	**h**
14	*Ubc*13:*U*:*Mms*2+*Rad*5:*PCNA*_on_:*U*	*Ubc*13:*U*:*Mms*2:*Rad*5:*PCNA*_on_:*U*	7.8 ×10^−2^	[25,36,58,62], this study	**i**
15	*Ubc*13:*U*:*Mms*2:*Rad*5:*PCNA*_on_:*U*	*Rad*5:*PCNA*_on_:*U*+*Ubc*13:*U*:*Mms*2	1 ×10^−10^	[25,36,58,62]	**i**
16	*Ubc*13:*U*:*Mms*2:*Rad*5:*PCNA*_on_:*U*	*Ubc*13:*Mms*2+*Rad*5:*PCNA*_on_:*U*:*U*	5 ×10^−2^	[36,54,63,64]	**j**, **k**
17	*Rad*5:*PCNA*_on_:*U*:*U*	*Rad*5+*PCNA*_on_:*U*:*U*	7.5 ×10^−6^	This study	**k**
18	*PCNA*_on_:*U*:*U*+*Rad*5	*Rad*5:*PCNA*_on_:*U*:*U*	5 ×10^−6^	This study	-
19	*Ubc*13:*U*:*Mms*2+*Rad*5:*PCNA*_on_:*U*:*U*	*Ubc*13:*U*:*Mms*2:*Rad*5:*PCNA*_on_:*U*:*U*	7.8 ×10^−2^	[25,36,58,62], this study	-
20	*Ubc*13:*U*:*Mms*2:*Rad*5:*PCNA*_on_:*U*:*U*	*Rad*5:*PCNA*_on_:*U*:*U*+*Ubc*13:*U*:*Mms*2	1 ×10^−10^	[25,36,58,62]	-
21	*Ubc*13:*U*:*Mms*2:*Rad*5:*PCNA*_on_:*U*:*U*	*Ubc*13:*Mms*2+*Rad*5:*PCNA*_on_:*U*:*U*:*U*	5 ×10^−3^	[36,54,63,64]	**l**
22	*Rad*5:*PCNA*_on_:*U*:*U*:*U*	*Rad*5+*PCNA*_on_:*U*:*U*:*U*	5 ×10^−3^	This study	**l**
23	*PCNA*_on_:*U*	*U*+*PCNA*_off_	3 ×10^−8^	This study	**g**
24	*PCNA*_on_:*U*:*U*	*U*+*U*+*PCNA*_off_	8 ×10^−4^	This study	**n**
25	*PCNA*_on_:*U*:*U*:*U*	*U*+*U*+*U*+*PCNA*_off_	5 ×10^−3^	This study	**m**

The mechanistic model for the PRR pathway, developed according to the stochastic formulation of chemical kinetics, consists of 25 reactions among 23 molecular species. Each reaction is described by a set of reagents and a set of products, and is characterized by a stochastic constant (*c*_i_, i=1, …, 25), here expressed in sec^-1^. The following notation was used in writing the reactions: (*i*) X + Y represents an interaction between the molecular species *X* and *Y*; (*ii*) X : Y describes a molecular complex between species *X* and *Y*. Data reported in the fifth column specify the literature references from which we retrieved the information used to define the corresponding reaction. Labels given in the last column associate each reaction to the main steps of the PRR pathway that are graphically represented in Figure 1.

All other stochastic constants were manually tuned by exploiting the time-courses of mono-, di- and tri-ubiquitylated PCNA isoforms derived from western blots of the experiments performed at 5 J/m^2^, considered as reference dose for parameter calibration. The choice of the initial parameterization at 5 J/m^2^ was then corroborated by comparing the outcome of stochastic simulations with the *in vivo* dynamics of PCNA ubiquitylation at 10 J/m^2^ UV dose.

#### Number of DNA lesions

The number of DNA lesions (CPD plus 6-4 PP) generated on the genome after different UV dose expositions was determined by using literature data [65,66]. These measurements, reported in Table 4, were exploited to derive the linear regression equations required to estimate the correlation between the radiation doses used in our laboratory experiments, and the corresponding number of lesions induced on *S. cerevisiae* genome.

**Table 4:** Experimental measurements of DNA lesions per genome at different UV irradiation doses

**UV irradiation dose (J/m**^**2**^**)**	**Number of lesions**	**Reference**
0.1	22	[66]
1	200	[65]
29	6000	[65]
108	24000	[66]
150	30000	[65]

In particular, we derived the equations *y*=200.248 *x* and *y*=222.22 *x*, by using data reported in [65,66], respectively (where *y* is the number of genomic lesions induced by the UV dose *x*). Afterwards, a *χ*^2^ cross-validation test was applied over the two datasets. The result of the cross-validation indicated that the best coefficient is the one related to the data reported in [65], being *χ*^2^ = 0.049 for the dataset from [65] and *χ*^2^ = 129.79 for the dataset from [66]. Therefore, the first equation was applied to estimate the number of genomic lesions induced by the various UV doses considered in our laboratory experiments. These values, reported in Table 5, represent an important input parameter for the mechanistic model of PRR, and are necessary to investigate its response under different UV irradiation conditions.

**Table 5:** Estimation of the number of DNA lesions per genome induced by different UV irradiation doses

**UV irradiation dose (J/m**^**2**^**)**	**Estimated number of lesions**
5	1001
10	2002
20	4005
30	6007
50	10012
75	15018

### Simulations setup

The model presented here was simulated and analyzed with the software BioSimWare [67]. All stochastic simulations were performed by using the tau-leaping algorithm [68], which represents one of the most efficient methods for simulating the temporal evolution of biochemical systems. Tau-leaping is an approximated but accurate version of the stochastic simulation algorithm (SSA) [28]; it allows to select and execute in parallel several reactions per step – instead of executing the reactions in a sequential manner, as it is done with SSA – thus speeding up the computation.

In particular, the efficiency of tau-leaping was exploited to carry out a parameter sweep analysis (PSA), with the aim of investigating the effect of varying the value of molecular amounts and the value of reaction constants on the dynamics of the PRR pathway. PSA was performed using a computational tool that generates a set of different initial conditions for the model and then automatically executes the corresponding stochastic simulations. With this tool, the value of each analyzed parameter varies within a specified range (with respect to a fixed reference value). To be more precise, the sweep analysis varies a single parameter from time to time, considering a linear (logarithmic, respectively) sampling of values within the specified range in the case of molecular amounts (reaction constants, respectively). The logarithmic sampling allows to uniformly span different orders of magnitude of the value of the chosen parameter using a reduced but fine-grained set of samples, therefore efficiently analyzing the dynamics of the system in a broad range of experimental conditions.

In this work, the PSA executed to check the reliability of the values of the stochastic constants and of the molecular amounts, that is, the parameterization used in the PRR model, was set as follows: 

•The value of each stochastic constant was varied of 3 orders of magnitude above and 3 below the reference value (given in Table 3);

•the value of the molecular amounts initially present in the system was varied in a range between 0 and twice the reference value (given in Table 2), thus mimicking the biological conditions ranging from the deletion to a 2-fold overexpression of the initial species.

In addition to PSA, we performed a global sensitivity analysis (SA) on the values of stochastic constants by exploiting a screening test called “method of the elementary effects” (EE), as described in [69,70]. This method allows to investigate how a specified model outcome changes according to a perturbation of the model input factors, realized by varying one input factor at a time while keeping all the others fixed. In this work, the variation interval of the input factors was defined over 4 orders of magnitude, 2 below and 2 above the reference value of each reaction constant (given in Table 3). As a result, the EE method associates to each input factor its so-called elementary effect, defined as the ratio between the variation in the model output and the variation in the input factor itself. The two model outputs considered for SA correspond to the molecular amounts of mono- and poly-ubiquitylated PCNA isoforms. More details on the SA method exploited in this work, as well as the ranking of the reactions according to their relevance within the model, are given in Additional file 3 and graphically represented in Additional file 4.

The results obtained from both PSA and SA confirmed the reliability of the chosen parameterization of the model, as shown further on.

All stochastic simulations were performed on a personal computer with a CPU Intel Core i5 M 520 @ 2.40 GHz, 4 GB RAM running Linux (Ubuntu 11.04). The mean duration time to execute one run of the tau-leaping algorithm to simulate the dynamics of the PRR model over 5 h (i.e., the time interval considered during laboratory experiments to measure the time-courses of PCNA ubiquitylation) is about 1 min for low UV doses and a dozen of minutes for high UV doses, using the initial values of molecular amounts given in Table 2 and the stochastic constants reported in Table 3.

### Representation of simulation outcomes and comparison with experimental data

The consistency of the PRR model was validated by comparing the outcome of stochastic simulations with the experimental measurements carried out on the wild type (WT) yeast strain at various UV doses. To this aim, by considering the western blots at each UV irradiation dose, we first quantified the values of mono-, di- and tri-ubiquitylated PCNA ratios (denoted by $÷{\text{PCNA}}_{\text{exp}}^{{\text{Ub}}_{\text{u}}}$, where *u*=1,2,3 corresponds to the three ubiquitylated isoforms), together with the respective mean *μ*($÷{\text{PCNA}}_{\text{exp}}^{{\text{Ub}}_{\text{u}}}$) and standard deviation *σ*($÷{\text{PCNA}}_{\text{exp}}^{{\text{Ub}}_{\text{u}}}$) of each PCNA isoform. This quantification is described in details in Additional file 2.

Then, from the outcome of stochastic simulations we derived the molecular amounts of PCNA isoforms (denoted by $\#{\text{PCNA}}_{\text{sim}}^{{\text{Ub}}_{\text{u}}}$, where *u*=1,2,3 corresponds to the three ubiquitylated isoforms). In particular, to tame the effect of stochastic fluctuations that are inherent in these computational analysis, we exploited the outcomes of a set of independent simulations (performed with the same initial conditions) to calculate the mean *μ*($\#{\text{PCNA}}_{\text{sim}}^{{\text{Ub}}_{\text{u}}}$) and standard deviation *σ*($\#{\text{PCNA}}_{\text{sim}}^{{\text{Ub}}_{\text{u}}}$) of PCNA amounts.

Afterwards, since we had to compare different kinds of measurements – namely, ratios of modified PCNA derived from laboratory experiments on the one side, and molecular amounts of modified PCNA obtained from stochastic simulations on the other side – we introduced two different strategies (see details in Additional file 5) for the graphical representation and comparison of the experimental and the computational results: 

1. The first strategy, called “normalized representation” (NR), consists of stacked bar graphs: for each sample analyzed within the time interval of 0-5 h, the stacked bars corresponding to the normalized ratios of mono-, di- and tri-ubiquitylated PCNA isoforms obtained from stochastic simulations (denoted by $÷{\text{PCNA}}_{\text{sim}}^{{\text{Ub}}_{\text{u}}}$) are plotted side by side to the experimental bars $÷{\text{PCNA}}_{\text{exp}}^{{\text{Ub}}_{\text{u}}}$ (which, as stated above, are already expressed as ratios);

2. in the second strategy, called “units representation” (UR), the molecular amounts derived from stochastic simulations $\#{\text{PCNA}}_{\text{sim}}^{{\text{Ub}}_{\text{u}}}$ are compared to the western blot quantifications which, in this case, were specifically transformed into molecular quantities (denoted by $\#{\text{PCNA}}_{\text{exp}}^{{\text{Ub}}_{\text{u}}}$).

We stress the fact that the NR allows a direct comparison between the experimental and simulation results, by considering the ratio of the three ubiquitylated isoforms of PCNA with respect to the total amount of modified PCNA measured in the system. Anyway, this strategy does not give any knowledge on the actual amount of modified PCNA, and it does not allow to clearly evidence the switch-off of PCNA ubiquitylation signal as long as the DNA lesions get processed, which can be instead directly represented by using the UR.

In what follows, we will use both NR and UR to give alternative representations of experimental measurements and simulations outcomes in WT and mutant yeast cells, in order to compare the variation of modified PCNA ratios, as well as to clearly display the dynamics of modified PCNA amounts.

## Results and discussion

In this section we present the results achieved from the integration between laboratory work and computational analysis, together with a discussion of the emergent issues concerning PRR. We start by presenting the identification of the spatio-temporal cascade of proteins association involved in PRR, as well as the stoichiometry of the corresponding protein complexes, which was performed through a structural modeling approach. This information was exploited, together with experimental data and the available knowledge on the molecular processes occurring during PRR, to define a novel mathematical model of PCNA ubiquitylation involved in the bypass of UV-induced DNA lesions.

Afterwards, we present the biological insights on PRR achieved from the comparison between laboratory experiments and stochastic simulations of the PRR model, at both low and high UV doses, and we discuss, in particular, the effects of the estimated number of UV-induced DNA lesions and of the intracellular levels of ubiquitin on the system dynamics. We show how a divergent behavior between wet data and computational outcomes at high UV doses led to the design of new laboratory experiments, that allowed us to suggest novel aspects on the functioning of PRR in living cells.

### Structural modeling of uncharacterized protein-protein complexes

Protein ubiquitylation is a multistep process carried out by the concerted action of activating (E1), conjugating (E2) and ligating (E3) enzymes, which can possibly support the generation of poly-ubiquitin chains [71]. In eukaryotes, all ubiquitin-associated pathways are characterized by a crescent complexity, since more E2s than E1s, and more E3s than E2s exist; therefore, the number of proteins potentially involved in each step increases, as well as the specificity of binding to the next substrate [72].

*In vitro* evidence [73] previously showed that the ubiquitylation reactions involve sequential E1-E2 and E2-E3 interactions, with E2 disengaging from E1 before it can interact with E3. This is in agreement with structural studies indicating that the E3 and E1 binding sites on E2s are partially overlapping [74-78].

In budding yeast, there is a unique E1 enzyme (Uba1), eleven E2 enzymes and more than fifty E3 enzymes [79], a few of which are directly involved in PRR. In order to clarify the spatio-temporal cascade of association among the enzymes involved in the mono- and poly-ubiquitylation of PCNA, as well as to deduce the stoichiometry of the respective protein complexes, we exploited a bioinformatic approach based on three-dimensional (3D) modeling to perform the reconstruction of the hypothetical structures of the E1-E2 and E2-E3 enzyme complexes involved in PRR.

To this aim, we considered the known 3D structures of proteins involved in PRR to deduce the 3D structure of their uncharacterized molecular complexes. More precisely, we exploited the published crystallographic structures of the proteins of our interest that were available on Protein Data Bank (PDB) and the PDB viewer software [80] (the PDB accession code for protein complexes analyzed in this work are listed in Additional file 6).

This approach confirmed that also in budding yeast the E1-E2 and E2-E3 complexes involved in PCNA ubiquitylation are likely mutually exclusive. More specifically, following the procedure described in [62,81], we constructed the E1-E2 and E2-E3 complexes involved in the mono-ubiquitylation of PCNA and in its poly-ubiquitylation through chain elongation. Our results suggest that: 

•The E1-E2 and E2-E3 complexes (Uba1-Rad6 and Rad6-Rad18, respectively) involved in PCNA mono-ubiquitylation are mutually exclusive (see Additional files 7 and 8) and that the mechanism of PCNA mono-ubiquitylation – from ubiquitin activation to its covalent linkage on PCNA – consists in a step-wise process, as also suggested by previously published results [73];

•the E1-E2 and E2-E3 complexes (Uba1-Ubc13 and Ubc13-Rad5, respectively) involved in PCNA poly-ubiquitylation are mutually exclusive (see Additional files 8 and 9). Moreover, the analysis of the hypothetical complex Uba1-Ubc13-Mms2 – involving the ubiquitin conjugating enzyme variant Mms2, which works together with Ubc13 – suggests that also the two complexes Uba1-Ubc13 and Ubc13-Mms2 are mutually exclusive: Ubc13 needs to be charged with ubiquitin by Uba1 before binding Mms2. Also in this case, our results support previous data [73], which argue for a distributive/step-wise sequence of events for PCNA poly-ubiquitylation, starting from ubiquitin activation to its covalent linkage on mono-ubiquitylated PCNA.

These results highlight the modularity of the whole process of PCNA ubiquitylation and allowed us to address the definition of the mathematical model of PRR on the most relevant biological process only, that is, PCNA ubiquitin conjugation. In particular, we could neglect the whole cascade of reactions involved in the ubiquitin activation process, and reduce to two simple reactions the detailed biochemical steps related to the ubiquitin trans-thio-esterification process, as described in the following section.

### The PCNA ubiquitylation model

#### Model assumptions

Wet laboratory experiments, combined with the knowledge built upon an accurate literature analysis, led us to the following major assumptions in the development of the model of PCNA ubiquitylation: 

1. A step-wise formation of the ubiquitin chain on PCNA;

2. a limited extension of the ubiquitin chain on PCNA;

3. a generic mechanism for the switch-off of the ubiquitylation signal.

These assumptions are motivated on the following bases: 

1. A step-wise formation of the ubiquitin chain is strongly motivated by *in vitro* assays [36]. In this context, the addition of a single unit of ubiquitin at a time for PCNA mono-ubiquitylation and K63-linked chain elongation, through multiple cycles of enzymatic catalysis mediated by E1, E2 and E3 enzymes, displays the following biochemical scheme for ubiquitin recruitment:

$$\begin{array}{cc} \;E1:U+E2\;\; & \to \;\;E1+E2:U\\ E3+\text{PCNA}:U_{i}\;\; & \to \;\;E3:\text{PCNA}:U_{i}\\ E2:U+E3:\text{PCNA}:U_{i}\;\; & \to \;\;E2:U:E3:\text{PCNA}:U_{i}\\ E2:U:E3:\text{PCNA}:U_{i}\;\; & \to \;\;E2:E3:\text{PCNA}:U_{i+1}\\ E2:E3:\text{PCNA}:U_{i+1}\;\; & \to \;\;E2+E3+\text{PCNA}:U_{i+1} \end{array}$$

where the classical formalism *X*+*Y* is used to denote an interaction between the generic molecular species *X* and *Y*, while *X*:*Y* denotes a molecular complex. Precisely, the notation *PCNA*:*U*_*i*_ comprehensively represents that a DNA lesion has been identified (considering for this purpose, by abuse of notation, the form *PCNA*:*U*_*i*_ for *i*=0), and describes as well the mono-ubiquitylated (for *i*=1) and poly-ubiquitylated (for *i*>1) isoforms of PCNA. Enzymes E2 and E3 correspond to Rad6 and Rad18 for mono-ubiquitylation, and to Ubc13 and Rad5 for poly-ubiquitylation, while E1 represents Uba1 in both cases. We remark that, in what follows, the effective role of enzyme E1 will not be formally considered, since the processes of ubiquitin activation and trans-thio-esterification can be widely reduced by deriving other simple reactions that describe the load of ubiquitin moieties on the E2 enzymes.

**Figure 2:**
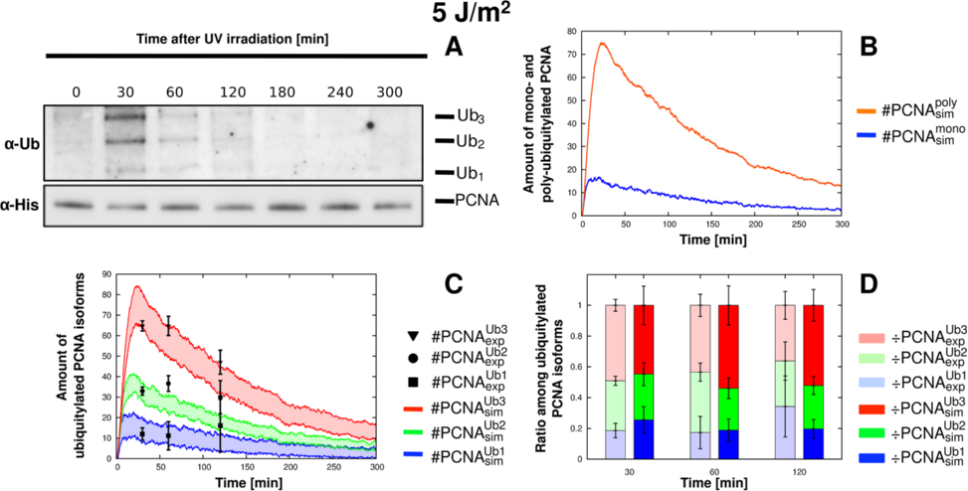
**Comparison between experimental and simulation results of PCNA ubiquitylation dynamics obtained on wild type yeast cells at 5 J/m**^**2**^** UV dose.** The figure shows the experimental measurements on WT yeast cells irradiated at 5 J/m^2^ UV dose and the comparison with the corresponding simulation results. (**A**) Representative image of a western blot showing a time-course measurement of mono-, di- and tri-ubiquitylated PCNA isoforms (top part, denoted by *α*-*Ub*) and of non modified PCNA (bottom part, denoted by *α*-*His*), sampled from 0 to 5 h after UV irradiation. The experiment was repeated 3 times. (**B**) Average dynamics of mono-ubiquitylated PCNA (blue line) and of poly-ubiquitylated PCNA (orange line), obtained from 100 independent stochastic simulations, executed starting from the same initial conditions (see Table 2 for molecular amounts and Table 3 for reaction constants) and with an estimated number of DNA lesions equal to 1001. (**C**) Comparison between the mean dynamics of mono-, di- and tri-ubiquitylated PCNA isoforms emerging from 100 independent stochastic simulations, and the mean value of experimental data $\mu (\#{\text{PCNA}}_{\text{exp}}^{{\text{Ub}}_{\text{u}}})$, together with the respective standard deviation $\sigma (\#{\text{PCNA}}_{\text{exp}}^{{\text{Ub}}_{\text{u}}})$. Colored areas indicate the amplitude of stochastic fluctuations around the mean value $\mu (\#{\text{PCNA}}_{\text{sim}}^{{\text{Ub}}_{\text{u}}})$. Data are plotted by using the units representation (see Additional file 5). (**D**) Comparison between the ratio of experimental ($÷{\text{PCNA}}_{\text{exp}}^{Ub_{u}}$, left bars) and simulated ($÷{\text{PCNA}}_{\text{sim}}^{{\text{Ub}}_{\text{u}}}$, right bars) ubiquitylated PCNA isoforms at every sampled time point where experimental measurements yield a detectable amount of modified PCNA. Mean and standard deviation bars of both experimental and simulated ratios are plotted by using the normalized representation (see Additional file 5).

**Figure 3:**
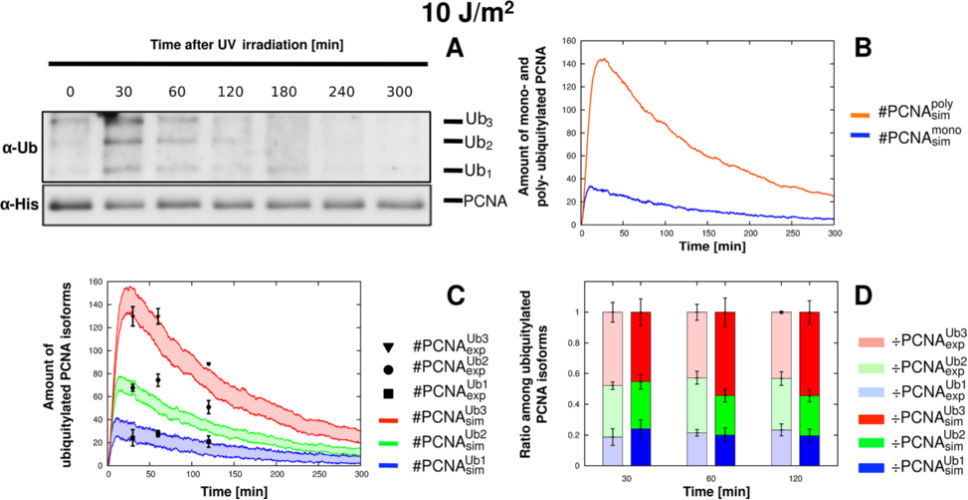
**Comparison between experimental and simulation results of PCNA ubiquitylation dynamics obtained on wild type yeast cells at 10 J/m**^**2**^** UV dose.** The figure shows the experimental measurements on WT yeast cells irradiated at 10 J/m^2^ UV dose and the comparison with the corresponding simulation results. (**A**) Representative image of a western blot showing a time-course measurement of mono-, di- and tri-ubiquitylated PCNA isoforms (top part, denoted by *α*-*Ub*) and of non modified PCNA (bottom part, denoted by *α*-*His*), sampled from 0 to 5 h after UV irradiation. The experiment was repeated 3 times. (**B**) Average dynamics of mono-ubiquitylated PCNA (blue line) and of poly-ubiquitylated PCNA (orange line), obtained from 100 independent stochastic simulations, executed starting from the same initial conditions (see Table 2 for molecular amounts and Table 3 for reaction constants) and with an estimated number of DNA lesions equal to 2002. (**C**) Comparison between the mean dynamics of mono-, di- and tri-ubiquitylated PCNA isoforms emerging from 100 independent stochastic simulations, and the mean value of experimental data $\mu (\#{\text{PCNA}}_{\text{exp}}^{{\text{Ub}}_{\text{u}}})$, together with the respective standard deviation $\sigma (\#{\text{PCNA}}_{\text{exp}}^{{\text{Ub}}_{\text{u}}})$. Colored areas indicate the amplitude of stochastic fluctuations around the mean value $\mu (\#{\text{PCNA}}_{\text{sim}}^{{\text{Ub}}_{\text{u}}})$. Data are plotted by using the units representation (see Additional file 5). (**D**) Comparison between the ratio of experimental ($÷{\text{PCNA}}_{\text{exp}}^{{\text{Ub}}_{\text{u}}}$, left bars) and simulated ($÷{\text{PCNA}}_{\text{sim}}^{{\text{Ub}}_{\text{u}}}$, right bars) ubiquitylated PCNA isoforms at every sampled time point where experimental measurements yield a detectable amount of modified PCNA. Mean and standard deviation bars of both experimental and simulated ratios are plotted by using the normalized representation (see Additional file 5).

2. The assumption of limiting to the third unit the elongation of the K63-linked ubiquitin chains on PCNA was taken because the detection of tetra-ubiquitylated PCNA is not technically reproducible. Moreover, N-ubiquitylated PCNA(*N*>4) rarely appears after DNA damage [38]. Notwithstanding this choice, simulation outcomes show the capability of our model to discriminate between the mono- and the poly-ubiquitylation contributions in the functioning of PRR, as discussed later on.

3. The last assumption is motivated by the fact that our laboratory experiments conducted at low doses of UV irradiation show the switch-off of PCNA ubiquitylation signal (see, e.g., the western blots in Figure 2A and Figure 3A), as also previously evidenced in [27]. This switch-off mechanism has not been precisely characterized yet. At present, there are no evidences for the activity of enzymes acting on PCNA deubiquitylation in yeast; however, even if we are not able to detect the real enzymatic mechanisms occurring *in vivo* in the PRR pathway, the results of our experimental setup report unmistakably the presence and the related global effect of the signal switch-off.

#### Definition of the mechanistic model

The information collected from literature, together with our assumptions, led to the development of the model of the PRR pathway depicted in Figure 1, and to the formulation of the biochemical reactions reported in Table 3. The mechanistic model consists of 23 molecular species (7 of which appear in the initial state of the system, as reported in Table 2) and 25 reactions, which can be clustered into four functional modules whose detailed description is reported hereby.

##### PCNA mono-ubiquitylation

The first module, consisting of reactions 1 to 10 in Table 3, describes the process of PCNA mono-ubiquitylation. Reaction 1 models the identification of the UV-induced lesion (denoted by *L*) by means of the replication machinery moving on the DNA strand and getting blocked by the lesion; this reaction is assumed to be non reversible. We denote by the symbol *PCNA*_on_ the PCNA clamp whenever the replication fork hits the DNA lesion (see also Figure 1, parts (b) and (c)). Reactions 2 and 3 illustrate the formation and separation of the Rad18 dimer, whereas reaction 4 resumes the simplified ubiquitin loading mechanism of Rad6, excluding the direct intervention of the E1 enzyme Uba1. Reactions from 5 to 10 describe the formation of the first ubiquitylated isoform of PCNA (*PCNA*_on_:*U*), due to the concerted action of the E2 Rad6 and E3 Rad18 enzymes, as described in the general biochemical scheme presented above (see also Figure 1, parts (d)-(f)).

##### PCNA di-ubiquitylation

The second module, consisting of reactions 11 to 17 in Table 3, describes the molecular mechanisms leading to the addition of the second ubiquitin moiety on the mono-ubiquitylated PCNA. Similarly to reaction 4, reaction 11 resumes the simplified ubiquitin loading mechanism of Ubc13-Mms2, excluding the direct intervention of the E1 enzyme Uba1. Reactions from 12 to 17 describe the formation of the di-ubiquitylated isoform of PCNA (*PCNA*_on_:*U*:*U*), due to the concerted action of the E2 Ubc13-Mms2 and E3 Rad5 enzymes, as described in the general biochemical scheme presented above (see also Figure 1, parts (h)-(k)). In particular, reaction 12 and its reverse 13 model the binding of the mono-ubiquitylated isoform (*PCNA*_on_:*U*) to the E3 Rad5, while reactions 14 and 15 give rise to the formation of the heterotrimer *Ubc*13:*U*:*Mms*2. Reactions 16 and 17 describe the dissociation of the complex *Ubc*13:*U*: *Mms*2:*Rad*5:*PCNA*_on_:*U* while the ubiquitin moiety is transferred to *PCNA*_on_:*U*, thus inducing the formation of *PCNA*_on_:*U*:*U*.

##### PCNA tri-ubiquitylation

The third module, consisting of reactions 18 to 22 in Table 3, describes the molecular mechanisms leading to the addition of the third ubiquitin moiety on the di-ubiquitylated PCNA, leading to *PCNA*_on_:*U*:*U*:*U* (see also Figure 1, dotted steps between parts (k) and (l)). Once more, these reactions resemble the biochemical scheme previously presented. In particular, reaction 18 models the capability of Rad5 to reassociate with the di-ubiquitylated isoform *PCNA*_on_:*U*:*U*, though we chose not to model the reverse reaction (equivalent to reaction 13, releasing Rad5 from *PCNA*_on_:*U*) to mark the biological difference between mono- and poly-ubiquitylation effects in PRR. Reactions 19-22 correspond to reactions 14-17, except for the fact that in this case *PCNA*_on_ carries two linked ubiquitin moieties instead of a single one.

##### Ubiquitylation signal switch-off

The fourth module, consisting of reactions 23 to 25 in Table 3, describes a generic mechanism for signal switch-off (denoted by *PCNA*_off_) of all three PCNA ubiquitylated isoforms, as suggested by experimental data and explained in our third assumption (see also Figure 1, parts (g), (m), (n)). These reactions also include a recycling mechanisms of ubiquitin moieties that, once released from PCNA, are made again available as input to the system and can be reloaded on Rad6 (reaction 4) and on *Ubc*13:*Mms*2 (reaction 13). We highlight here that these two reactions represent an important part of the model, as they influence the sensitivity of the model to free ubiquitin amounts, as discussed later on.

A previous definition of the model included an additional module describing the formation and disassociation of *Ubc*13:*U*:*Mms*2 and *Ubc*13:*Mms*2 complexes. After a careful verification that the system dynamics was not affected by the removal of this module, we chose to substitute this set of reactions with a congruous initial amount of the complex *Ubc*13:*Mms*2 (assumed to be already formed and available in the initial state of the system), in order to speed up the simulations. The initial amount of this complex was set to the minimum value between the amounts of the two proteins (Ubc13, Mms2) necessary for its formation, as reported in Table 2. For the sake of completeness, the set of removed reactions and the comparison of the simulated dynamics obtained by using the two versions of the model are reported in Additional file 10.

The SBML version of the model is available at the BioModels database [82,83] under submission identifier MODEL1211260000.

### Kinetics of PCNA ubiquitylation at low and high doses of UV irradiation

The levels of mono-, di- and tri-ubiquitylated PCNA measured *in vivo* after UV irradiation of WT yeast cells were used as the biological readout to validate the mechanistic model of PRR. As a matter of fact, the generally accepted PRR model assumes that K164 mono-ubiquitylation is a marker of the PRR error-prone/error-free TLS sub-pathway, while K164 di- and tri-ubiquitylation are a marker of the PRR error-free TS sub-pathway [25]. In order to derive time-series measurements of all ubiquitylated PCNA isoforms at the same time points and in the same yeast cells, we developed an experimental protocol that allows to detect both mono- and poly-ubiquitylated PCNA isoforms on a single western blot. This method represents indeed a relevant advantage with respect to other protocols previously devised for PCNA ubiquitylation [27], which could only allow the measurements of di- to N-ubiquitylated isoforms on the same film, without the mono-ubiquitylated one.

In [84], it was shown that physiological UV-induced responses of PRR are obtained by exposing cells to Chronic-Dose of UV light (CLUV) for 6-9 h (0.18 J/m^2^ min−1). However, we did not conducted laboratory experiments under these conditions since our experimental resolution does not allow the detection of PCNA ubiquitylation in chronically UV irradiated yeast cells. Indeed, in our laboratory experiments these irradiation doses are below the threshold for the detection of PCNA ubiquitylation; on the contrary, with our method the ubiquitylation signal becomes measurable at *acute* treatment with UV doses of 5 J/m^2^, at least, because of a technical limit of detection of the western blot technique. For this reason, we produced western blot time-courses as previously described by exposing cells either to low UV doses (5 and 10 J/m^2^) or to high UV doses (50 and 75 J/m^2^).

Concerning the experiments at the lower UV irradiation doses, the PCNA ubiquitylation signal in response to 5 J/m^2^ undergoes a quick increase, reaching its maximal value already 3 minutes after UV irradiation (Additional file 11), and starts to decrease after 30 minutes (Figure 2A). Afterwards, the signal intensity decreases, resulting barely detectable after 2 and 3 h because of the signal switch-off. Moreover, at 5 J/m^2^ the system is characterized by an intense PCNA poly-ubiquitylation signal, with respect to the mono-ubiquitylated PCNA isoform, which results in the activation of the TS sub-pathway. This behavior is well reproduced by means of computational simulations, which mimic the PRR functioning in response to an estimated number of 1001 lesions (Table 5). The match between experimental and computational results is clearly shown in Figure 2B, where we plot the dynamics of mono- and poly-ubiquitylated PCNA. Moreover, the simulated amounts of mono-, di- and tri-ubiquitylated PCNA isoforms well fit the experimental data, as reported in Figure 2C, where the average dynamics of the three PCNA isoforms is compared with the experimental measures, plotted by using the units representation (UR) (as explained in Additional file 5). This can also be seen in Figure 2D, where we compare the normalized stacked bars of the ratio of ubiquitylated PCNA isoforms obtained from stochastic simulations with those measured through wet experiments (here plotted using the normalized representation (NR), as explained in Additional file 5).

Analogous results were obtained at a UV dose of 10 J/m^2^, corresponding to an estimated number of 2002 lesions in the model (Table 5), as shown in Figure 3. These analyses indicate that the computational model is able to correctly reproduce the *in vivo* dynamics of PCNA ubiquitylation at low UV doses. In particular, the model accurately reproduces the experimental ratio between mono- and poly-ubiquitylated PCNA (Figure 2B and Figure 3B), which corresponds to the activity of the potentially mutagenic lesion bypass sub-pathway and of the error-free lesion bypass sub-pathway, respectively. In addition, the model correctly reproduces the switching-off of the UV lesion bypass signal at low UV doses.

The response of the model at low UV doses (namely, 10 J/m^2^) was also analyzed through a global SA, performed by considering the reaction constants as the input factors of the model, and through a PSA, carried out on the values of all reaction constants and of all initial molecular amounts. The most interesting results of these analyses are presented in Additional files 3 and 4 for SA, 12, 13, 14 and 15 for PSA.

**Figure 4:**
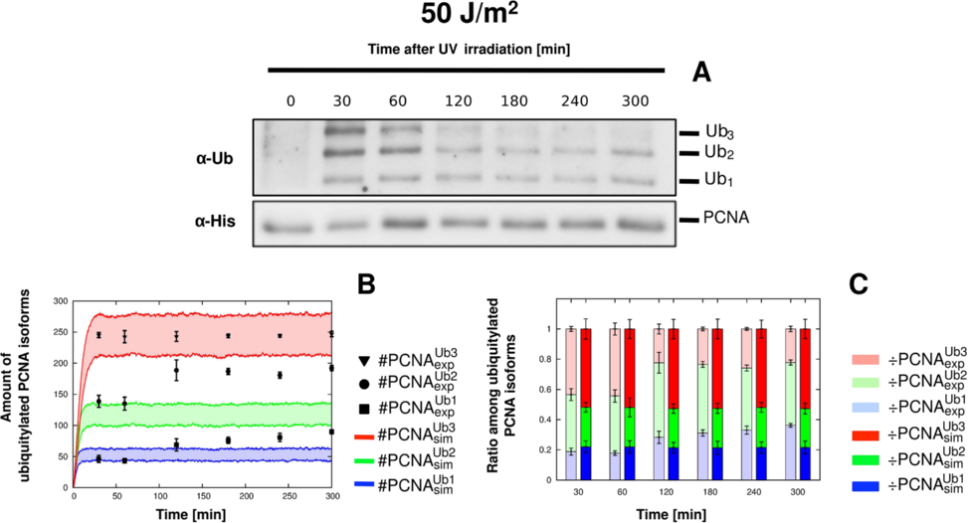
**Comparison between experimental and simulation results of PCNA ubiquitylation dynamics obtained on wild type yeast cells at 50 J/m**^**2**^** UV dose.** The figure shows the experimental measurements on WT yeast cells irradiated at 50 J/m^2^ UV dose and the comparison with the corresponding simulation results. (**A**) Representative image of a western blot showing a time-course measurement of mono-, di- and tri-ubiquitylated PCNA isoforms (top part, denoted by *α*- *Ub*) and of non modified PCNA (bottom part, denoted by *α*-*His*), sampled from 0 to 5 h after UV irradiation. The experiment was repeated 3 times. (**B**) Comparison between the mean dynamics of mono-, di- and tri-ubiquitylated PCNA isoforms emerging from 100 independent stochastic simulations, and the mean value of experimental data $\mu (\#{\text{PCNA}}_{\text{exp}}^{{\text{Ub}}_{\text{u}}})$, together with the respective standard deviation $\sigma (\#{\text{PCNA}}_{\text{exp}}^{{\text{Ub}}_{\text{u}}})$. Stochastic simulations were executed starting from the same initial conditions (see Table 2 for molecular amounts and Table 3 for reaction constants) and with an estimated number of DNA lesions equal to 10012. Colored areas indicate the amplitude of stochastic fluctuations around the mean value $\mu (\#{\text{PCNA}}_{\text{sim}}^{{\text{Ub}}_{\text{u}}}$). Data are plotted by using the units representation (see Additional file 5). (**C**) Comparison between the ratio of experimental ($÷{\text{PCNA}}_{\text{exp}}^{{\text{Ub}}_{\text{u}}}$, left bars) and simulated ($÷{\text{PCNA}}_{\text{sim}}^{{\text{Ub}}_{\text{u}}}$, right bars) ubiquitylated PCNA isoforms at every sampled time point. Mean and standard deviation bars of both experimental and simulated ratios are plotted by using the normalized representation (see Additional file 5).

**Figure 5:**
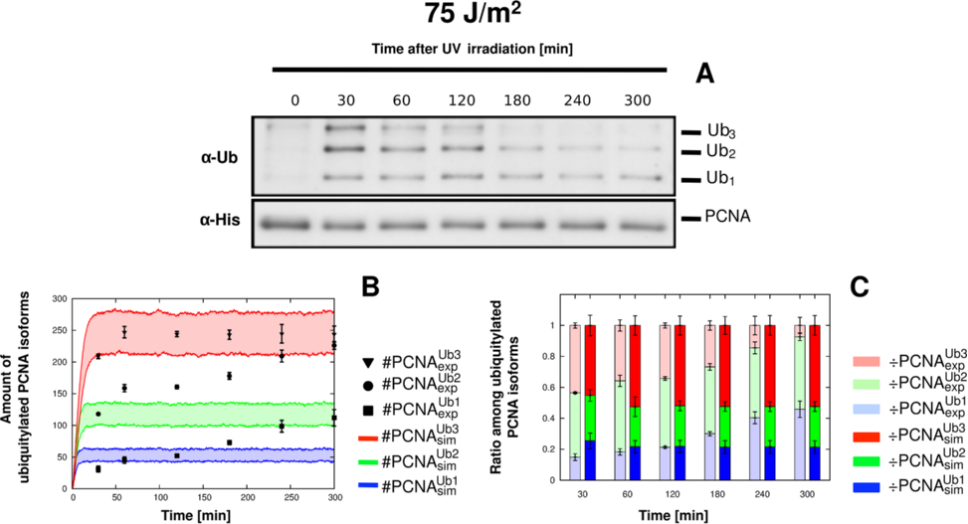
**Comparison between experimental and simulation results of PCNA ubiquitylation dynamics obtained on wild type yeast cells at 75 J/m**^**2**^** UV dose.** The figure shows the experimental measurements on WT yeast cells irradiated at 75 J/m^2^ UV dose and the comparison with the corresponding simulation results. (**A**) Representative image of a western blot showing a time-course measurement of mono-, di- and tri-ubiquitylated PCNA isoforms (top part, denoted by *α*- *Ub*) and of non modified PCNA (bottom part, denoted by *α*-*His*), sampled from 0 to 5 h after UV irradiation. The experiment was repeated 3 times. (**B**) Comparison between the mean dynamics of mono-, di- and tri-ubiquitylated PCNA isoforms emerging from 100 independent stochastic simulations, and the mean value of experimental data $\mu (\#{\text{PCNA}}_{\text{exp}}^{{\text{Ub}}_{\text{u}}})$, together with the respective standard deviation $\sigma (\#{\text{PCNA}}_{\text{exp}}^{{\text{Ub}}_{\text{u}}})$. Stochastic simulations were executed starting from the same initial conditions (see Table 2 for molecular amounts and Table 3 for reaction constants) and with an estimated number of DNA lesions equal to 15018. Colored areas indicate the amplitude of stochastic fluctuations around the mean value $\mu (\#{\text{PCNA}}_{\text{sim}}^{{\text{Ub}}_{\text{u}}})$. Data are plotted by using the units representation (see Additional file 5). (**C**) Comparison between the ratio of experimental ($÷{\text{PCNA}}_{\text{exp}}^{{\text{Ub}}_{\text{u}}}$, left bars) and simulated ($÷{\text{PCNA}}_{\text{sim}}^{{\text{Ub}}_{\text{u}}}$, right bars) ubiquitylated PCNA isoforms at every sampled time point. Mean and standard deviation bars of both experimental and simulated ratios are plotted by using the normalized representation (see Additional file 5).

Concerning the experiments at the higher, non physiological UV doses of 50 J/m^2^ and 75 J/m^2^, we observed a divergence between *in vivo* PCNA ubiquitylation measurements and the computational outcomes. At these UV doses, the simulated dynamics of mono-, di- and tri-ubiquitylated PCNA PCNA reach a stable steady state (see Figure 4B-C for 50 J/m^2^, Figure 5B-C for 75 J/m^2^), corresponding to a saturation-like trend. This is in contrast with the observed *in vivo* measurements, where we can evidence a dose-dependent increase of the mono-ubiquitylated PCNA isoform with respect to the sum of di- and tri-ubiquitylated PCNA isoforms (see Figure 4A for 50 J/m^2^, Figure 5A for 75 J/m^2^), reaching about 50% of the bypass signal after 5 h at 75 J/m^2^. This dose-dependent increase of mono-ubiquitylated PCNA is indeed biologically relevant, since it might correlate with the UV dose-dependent induced mutagenesis that was previously observed in [85].

After an extensive and careful verification that this divergent behavior was not due to the model layout (for either the topological structure of molecular interactions or the chosen parameterization), we hypothesized that the difference between the experimental and the computational outcomes might be due to an overestimation of the number of bypassed UV lesions at 50 J/m^2^ and 75 J/m^2^ UV doses, corresponding to 10012 and 15018 lesions, respectively. To clarify the reason why we obtained a divergent behavior of the model at low and high UV doses, we designed further laboratory experiments, as discussed in the next sections.

### Determination of UV dose-dependent threshold for the validation of the PRR model

To test *in vivo* the possible overestimation of the number of DNA lesions actually processed by PRR at high UV doses, we performed a time-course experiment using a yeast strain carrying a deletion of the *RAD14* gene (*rad14δ* strain, see Table 1). This gene codifies for a well-characterized protein of the NER pathway, which is responsible for the repair, rather than the bypass, of UV-induced lesions in the genome. It is well established that the deletion of this master NER gene in yeast essentially abrogates excision and repair of UV lesions by NER in the genome [86]. Therefore, by inactivating NER all DNA lesions should be processed by other response mechanisms to UV-induced damage, including PRR, and we should be able to test whether the *in vivo* dynamics of PCNA ubiquitylation in these saturating conditions match the computational dynamics obtained at high UV doses.

**Figure 6:**
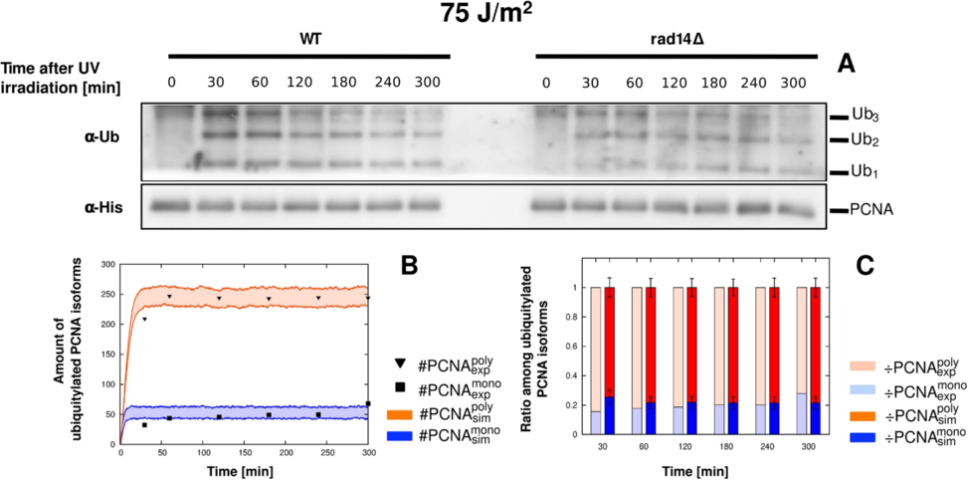
**Prediction of bypassed DNA lesions overestimation and validation results on*rad14Δ* background yeast cells at 75 J/m**^**2**^** UV dose.** The figure shows the experimental measurements on *rad14δ* background yeast cells irradiated at 75 J/m^2^ UV dose and the comparison with the corresponding simulation results. (**A**) Western blots showing a comparison between time-course measurements executed on WT cells (left part) and *rad14δ* background cells (right part), of mono-, di- and tri-ubiquitylated PCNA isoforms (top part, denoted by *α*- *Ub*) and of non modified PCNA (bottom part, denoted by *α*-*His*), sampled from 0 to 5 h after UV irradiation. As the aim of this experiment was not to carry out a precise quantification of the PCNA ubiquitylated isoforms, but only to verify the prediction of computational analysis, it was conducted with a single repetition. (**B**) Comparison between the values of experimental data ($\#{\text{PCNA}}_{\text{exp}}^{\text{mono}}$, $\#{\text{PCNA}}_{\text{exp}}^{\text{poly}}$) and the mean dynamics ($\#{\text{PCNA}}_{\text{sim}}^{\text{mono}}$, $\#{\text{PCNA}}_{\text{sim}}^{\text{poly}}$) of mono- and poly-ubiquitylated PCNA isoforms emerging from 100 independent stochastic simulations, where $\#{\text{PCNA}}_{\text{exp}}^{\text{poly}}$, $\#{\text{PCNA}}_{\text{sim}}^{\text{poly}}$ represent the sum of mono-, di- and tri-ubiquitylated PCNA amounts obtained from experimental data and simulation outcomes, respectively. Stochastic simulations were executed starting from the same initial conditions (see Table 2 for molecular amounts and Table 3 for reaction constants) and with an estimated number of DNA lesions equal to 15018. Colored areas indicate the amplitude of stochastic fluctuations around the mean values $\mu (\#{\text{PCNA}}_{\text{sim}}^{\text{mono}})$, $\mu (\#{\text{PCNA}}_{\text{sim}}^{\text{poly}})$. Data are plotted by using the units representation (see Additional file 5). (**C**) Comparison between the ratio of experimental ($÷{\text{PCNA}}_{\text{exp}}^{\text{mono}}$ and $÷{\text{PCNA}}_{\text{exp}}^{\text{poly}}$, left bars) and simulated ($÷{\text{PCNA}}_{\text{sim}}^{\text{mono}}$ and $÷{\text{PCNA}}_{\text{sim}}^{\text{poly}}$, right bars) ubiquitylated PCNA isoforms at every sampled time point. Mean and standard deviation bars of both experimental and simulated ratios are plotted by using the normalized representation (see Additional file 5).

As shown in Figure 6A, *RAD14* deletion causes a clear modification of the *in vivo* dynamics of PCNA ubiquitylation with respect to the WT strain at a UV dose of 75 J/m^2^. Indeed, in *rad14δ* cells we observed a dramatic decrease in the intensity of the signal, and we obtained a ratio between mono- and poly-ubiquitylated PCNA isoforms that well matches the computational results (Figure 6B-C). This validation experiment therefore supports our hypothesis that the computational model cannot properly reproduce the measured PRR response *in vivo* at high UV doses because of an overestimation of the bypassed DNA lesions.

**Figure 7:**
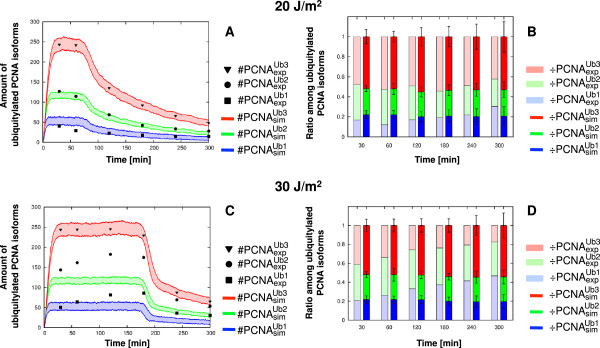
**Prediction of UV dose-dependent threshold and validation results on wild type yeast cells at 20 J/m**^**2**^** and 30 J/m**^**2**^** UV doses.** The figure shows the experimental measurements on WT cells irradiated at 20 J/m^2^ UV dose (top part) and at 30 J/m^2^ UV dose (bottom part), as well as the comparison with the corresponding simulation results. As the aim of these experiments was not to carry out a precise quantification of the PCNA ubiquitylated isoforms, but only to verify the prediction of computational analysis, they were conducted with a single repetition. (**A-C**) Comparison between the value of western blot quantification $\#{\text{PCNA}}_{\text{exp}}^{{\text{Ub}}_{\text{u}}}$ deriving from a single experiment, and the dynamics of mono-, di- and tri-ubiquitylated PCNA isoforms $\#{\text{PCNA}}_{\text{sim}}^{{\text{Ub}}_{\text{u}}}$ emerging from 100 independent stochastic simulations, executed starting from the same initial conditions (see Table 2 for molecular amounts and Table 3 for reaction constants), with an estimated number of DNA lesions equal to 4005 (**A**) and 6007 (**C**). Colored areas indicate the amplitude of stochastic fluctuations around the mean value $\mu (\#{\text{PCNA}}_{\text{sim}}^{{\text{Ub}}_{\text{u}}})$. Data are plotted by using the units representation (see Additional file 5). (**B-D**) Comparison between the ratio of experimental ($÷{\text{PCNA}}_{\text{exp}}^{{\text{Ub}}_{\text{u}}}$, left bars) and simulated ($÷{\text{PCNA}}_{\text{sim}}^{{\text{Ub}}_{\text{u}}}$, right bars) ubiquitylated PCNA isoforms at every sampled time point. Mean and standard deviation bars of simulated results are plotted by using the normalized representation (see Additional file 5).

As a consequence, considering the result obtained on the *rad14δ* strain and taking into account the different behaviors of the model at low and high UV doses in the WT strain, we tried to identify the UV dose threshold which ensures the proper functioning of the model. To this aim, we carried out additional wet experiments on WT strain cells irradiated at UV doses between 10 J/m^2^ and 50 J/m^2^, in order to detect the UV-dose dependent threshold that ensures a good match between *in vivo* measurements and computational results. As shown in Figure 7A-B, the model reproduces a proper dynamics at 20 J/m^2^, while its behavior starts to diverge from *in vivo* measurements at 30 J/m^2^ (Figure 7C-D). Therefore, we can conclude that at UV doses below 30 J/m^2^ our model is capable to mimic *in vivo* data or, stated otherwise, it is able to correctly describe the mono- and poly-ubiquitylation processes of PCNA taking place in the PRR pathway *in vivo*.

So, the next question we asked ourselves was: what kind of processes are actually occurring in living yeast cells, that are able to induce this contrasting behavior responses at acute low and high UV irradiation doses?

### Crosstalk between PRR and NER

As previously discussed, the trend of steady state dynamics of mono- and poly-ubiquitylated PCNA obtained with stochastic simulations resembles the biochemical kinetics of saturation, meaning that all PCNA molecules occurring in the system get involved in the lesion bypass processes. Therefore, we hypothesized a saturation of PRR *in vivo* because of an overestimation of the number of processed lesions in our experimental system. We reproduced this saturation *in vivo* by deleting the *RAD14* gene, which dramatically affected the dynamics of PCNA ubiquitylation after UV irradiation *in vivo*. More precisely, in NER-deficient cells we obtained, *in vivo*, a comparable steady state of mono- and poly-ubiquitylated PCNA species with respect to the computational results at high UV doses.

Altogether, these findings highlight a poorly characterized crosstalk between PRR and NER. Indeed, an optimal lesion bypass PRR activity, at least correlated to PCNA ubiquitylation, seems to depend on NER functionality. Therefore, to better characterize the effects of NER on UV-induced damage, we also evaluated whether its role might be important for proper S phase progression. It was shown that UV irradiation with 5 J/m^2^ of a G1 arrested *rad14δ* strain causes a cell cycle block at the G1/S transition [29]. When we UV irradiated (10 J/m^2^) a S phase synchronized *rad14δ* cell population, the lack of NER strongly impaired correct S phase progression, suggesting an underestimated function of NER also during S phase (see Additional file 16).

A possible explanation of our findings is that, on one side, we are overestimating the number of lesions bypassed by PRR in the computational model while, on the other side, *in vivo* PRR needs the contribution of the NER pathway to work properly. Considering such experimental data, it is likely that the number of lesions, which represents an important input parameter of our model, is actually dependent on the action of NER and on the combined crosstalk between these two pathways. Unfortunately, it is presently impossible to measure the exact number of lesions processed by NER and PRR *in vivo*, but we are currently working on a bioinformatic strategy based on DNA sequence analysis to predict the correct number of lesions to be used as input value of our model at any given UV irradiation dose, in a similar way to the approach presented in [87].

### Influence of ubiquitin amount

**Figure 8:**
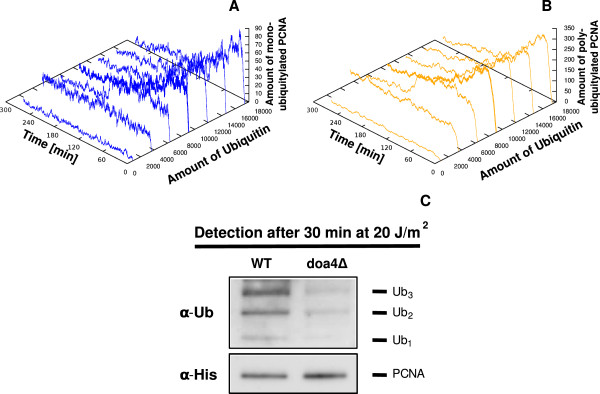
**Influence of free ubiquitin concentration and validation results on*doa4Δ* background yeast cells at 20 J/m**^**2**^** UV dose.** The figure shows the simulated dynamics of PCNA mono-ubiquitylation (**A**) and poly-ubiquitylation (**B**) at a UV dose of 20 J/m^2^, obtained from a PSA executed on the initial amount of ubiquitin, which is varied in the interval [870, 17396] molecules – mimicking the biological conditions ranging from a 10-fold reduction (corresponding to the severely impaired condition of *doa1δ* yeast cells) to a 2-fold overexpression of the total amount of free ubiquitin in WT cells (see the reference value in Table 2). In the plots, the thick lines correspond to the dynamics obtained with the reference value for ubiquitin amount. The simulations show that for ubiquitin amounts lower than the reference value, the amounts of mono- and poly-ubiquitylated PCNA decrease, as also observed experimentally in *doa4δ* cells (**C**, right part). On the other hand, by increasing the ubiquitin amount occurring in the system, the dynamics show an initial peak in the amount of mono- and poly-ubiquitylated PCNA, suggesting that high amounts of ubiquitin might lead the system to a faster bypass of all lesions with respect to the physiological reference value. (**C**) Western blot showing a comparison between time-course measurements in WT yeast cells (left part) and *doa4δ* yeast cells (right part) of the mono-, di- and tri-ubiquitylated PCNA isoforms (top part, denoted by *α*-*Ub*) and of non modified PCNA (bottom part, denoted by *α*-*His*), sampled at 30 min after UV irradiation. As the aim of these experiments was not to carry out a precise quantification of the PCNA ubiquitylated isoforms, but only to verify the prediction of computational analysis, they were conducted with a single repetition.

As a further investigation of the functioning of the PRR pathway, we tested the influence of ubiquitin amount on the performance of the computational model and the effect of *in vivo* reduction of free ubiquitin amount. In living cells, ubiquitin is usually kept at stable levels through homeostatic mechanisms. The main actors in this process are deubiquitylating enzymes (DUBs), which recycle ubiquitin moieties from ubiquitylated proteins. The reduction of free ubiquitin in the cell can potentially impair PRR as well as all ubiquitin-related pathways, such as protein degradation/proteasome, cell cycle, DNA repair, chromatin remodeling, etc. Therefore, it is likely that the free cellular level of ubiquitin could act as a limiting factor for PRR, given the competition with other molecular processes. To verify this hypothesis, we carried out a PSA to explore the influence of the level of free ubiquitin on PCNA ubiquitylation dynamics. The simulation results evidenced that the model is sensitive to this variation, as shown in Figure 8A-B, which reports the dynamics of PCNA mono-ubiquitylation and poly-ubiquitylation obtained from a PSA carried out on the initial amount of ubiquitin. As clearly shown in the plots, for amounts of ubiquitin lower than the reference value (around 8700 molecules/cell, see Table 2), the amounts of mono- and poly-ubiquitylated PCNA isoforms decrease. On the other hand, by increasing the number of ubiquitin molecules present inside the system, the dynamics show an initial peak in the number of mono- and poly-ubiquitylated PCNA molecules, suggesting that higher amounts of free available ubiquitin might lead to an increase in PCNA ubiquitylation, possibly influencing PRR.

The possible occurrence of similar effects *in vivo* was investigated through *ad hoc* experiments in living cells. Indeed, it was previously shown that the deletion of *DOA1*, a gene coding for a DUB essential for ubiquitin homeostasis, causes a dramatic reduction of the free ubiquitin pool in budding yeast log phase cells [88]. This reduction correlates with sensitivity to DNA damaging and replication stress agents (such as UV, methyl methanesulfonate (MMS), hydroxyurea) and abolishes PCNA ubiquitylation in the presence of MMS [30,88].

Since the reduction of free ubiquitin in *doa1δ* cells is so strong to impair both sensitivity to UV damage and PCNA ubiquitylation after DNA damage, we tested a less extreme *in vivo* situation. Namely, we considered *DOA4*, a gene coding for another DUB whose deletion was shown to cause, in log phase yeast cells, a 3-fold reduction of free ubiquitin and a weak sensitivity to UV-induced damage [30,89,90]. We thus tested *in vivo* the effect of *DOA4* deletion on the dynamics of PCNA ubiquitylation at 20 J/m^2^, a dose compatible with the threshold under which our model behaves properly (Figure 7A-B). As shown in Figure 8C, the deletion of *DOA4* and the related reduction of the ubiquitin pool cause *in vivo* a reduction of about 65% of PCNA ubiquitylation in our *doa4δ* yeast strain. This is in agreement with our computational analysis, as shown in Figure 8A-B, which reports the dynamics of PCNA mono-ubiquitylation and poly-ubiquitylation obtained at a UV dose of 20 J/m^2^.

We can therefore conclude that the level of free ubiquitin occurring in the system is one of the most sensitive parameters of the PRR model, and suggests that the ubiquitin pool needs to be actively maintained at a constant level since any change in its intracellular concentration has a large influence in downstream processes. This is in agreement both with previous results and with our model-driven experimental verification on *doa4δ* yeast strain.

## Conclusions

In this paper we propose a novel computational model describing the PRR pathway in *S. cerevisiae*, involved in UV-induced DNA damage bypass. As wet readout of PRR activity in wild type and mutant yeast cells, in response to different doses of UV irradiation, we considered the intracellular levels of mono-, di- and tri-ubiquitylated PCNA on the K164 residue. In fact, the generally accepted biological model of PRR assumes that K164 mono-ubiquitylation is a marker of the PRR error-prone/error-free TLS sub-pathway, while K164 di- and tri-ubiquitylation are a marker of the PRR error-free TS sub-pathway [25]. We realize that this is an indirect estimate of PRR and further experiments will be required to measure directly TLS and TS.

The comparison between experimental measurements and computational outcomes showed that our model correctly describes the functioning of PRR response at UV doses lower than 30 J/m^2^, approximately. On the contrary, at higher UV doses the dynamics of PCNA ubiquitylation obtained from computational simulations is characterized by a quick saturation, reaching a stable steady state for all the analyzed PCNA isoforms. In the attempt to better understand these results, we found that NER, the repair pathway known to fix UV-induced lesions during the G1 (see [91], Figure S1-C) and G2 [92] phases of the cell cycle, is required also for a proper S phase progression in response to UV irradiation.

This NER connection suggests intricate functional crosstalks between PRR and other pathways controlling genome stability. Indeed, in addition to NER, PRR was shown to be functionally linked to homologous recombination genes [93] and to the DNA damage checkpoint, which seems to affect the error-free sub-pathway but not the error-prone sub-pathway [94]. Moreover, it was recently found that defects in DNA ligase I (codified by *CDC9* gene) leads to mono-ubiquitylation of PCNA on the K107 residue, rather than on K164 [95]. This PCNA modification requires the E2 variant Mms2 in conjunction with Ubc4 and the E3 Rad5, and occurs before full checkpoint activation [95]. Accumulation of DNA nicks in response to high UV doses and saturation of other downstream actors of PRR, such as DNA ligase I, may also cause K107 ubiquitylation after the DNA damage-specific ubiquitylation on K164. We might therefore speculate that, in the PCNA homotrimer, each monomer can be modified at least on two different residues at the same time, by different modifiers. The PRR pathway can thus be more complicated and less far characterized than previously thought. We are presently working on the experimental characterization of these new PRR aspects, with the aim of gaining new biological insights into the effective functioning of PRR *in vivo*, and of retrieving additional information to improve the computational model presented here.

Another intriguing aspect that we predicted by means of computational analysis and then verified by means of *ad hoc* designed experiments, is the relevance of ubiquitin amount on the DNA damage response in yeast and, in particular, on the PRR pathway. Ubiquitin is usually kept at stable levels through homeostatic mechanisms involving DUBs, which recycle ubiquitin moieties from ubiquitylated proteins. The reduction of free ubiquitin in the cell can potentially impair all ubiquitin-related pathways. Indeed, deletion of some DUBs in budding yeast causes a UV sensitivity that seems to correlate with the extent of free ubiquitin reduction [30,88]. We thus explored the effect of variations in the level of free ubiquitin in PCNA ubiquitylation dynamics, through a parameter sweep analysis. We found that the model is sensitive to variation of free ubiquitin amounts; in particular, a 3-fold reduction of free ubiquitin, obtained *in vivo* by deleting the *DOA4* gene, causes a 65% reduction of PCNA ubiquitylation in response to 20 J/m^2^ UV irradiation.

An aspect of PRR that is still to be elucidated concerns the fate of K164 mono- and poly-ubiquitylated PCNA after its activity on DNA damage bypass. Through dedicated laboratory experiments we tried many solutions to inhibit the hypothetical yet still unknown steps of signal switch-off; for instance, we carried out the deletion of the RCF1-like proteins (Rad24, Elg1 and Ctf18), in order to block the unloading of PCNA from chromatin, as well as of other DUBs like Ubp15 – the homologous of the specific PCNA DUBs Ubp21 and Ubp22 in *Schizosaccharomyces pombe*[96] – and Ubp10. We confirmed that Ubp10 participates to PCNA deubiquitylation, as reported by [97]. However, deletion of both *UBP10* and *UBP15* did not prevent the disappearance of ubiquitylated PCNA at later time-points in the conditions tested (Additional file 17). This finding indicates that additional mechanisms likely cooperate in switching-off PCNA ubiquitylation signaling. Whenever novel insights will be learned in relation to the fate of ubiquitylated PCNA in response to UV-induced damages, a further refinement of our computational model could be performed, in order to describe in details the molecular interactions involved in the effective mechanism of the ubiquitylation signal switch-off.

Finally, a further challenging aspect emerging from our analysis is the presence of the slight discrepancy between the experimental and computational ratios of di- and tri-ubiquitylated PCNA isoforms found, e.g., at 10 J/m^2^. This could be due to an overestimation of the number of bypassed lesions, as previously discussed, but another and more interesting hypothesis to explain this finding is the possible presence of two different modifications on a single monomer of PCNA at the same time. In fact, PCNA can be covalently modified also on K127 residue by SUMO [98] and on K107 by ubiquitin [95]. At the present moment, we cannot exclude the possibility of different combinations of simultaneous modifications of PCNA, which give origin to distinct hybrid molecules; however, since these complexes are characterized by the same molecular mass, it is hard to distinguish them with standard laboratory techniques. This hypothesis and the novel biological insights gained with our Systems Biology approach indeed open new research perspectives on PRR, that are worth to be thoroughly investigated.

In conclusion, we used a combination of genetic, biochemical, structural and computational approaches to investigate the molecular mechanisms of PCNA ubiquitylation involved in the activation of the PPR pathway *in vivo*. PRR mechanisms are well conserved from yeast to man and it is well established that PRR defects are linked to increased genome instability and cancerogenesis. The original computational model of PRR presented here might be extended in the future to other eukaryotic cells by integrating novel knowledge coming from further experimental data, or used as a basic component within a modular computational approach to analyze the crosstalk with other pathways involved in genome stability.

## Abbreviations

6-4 PP: 6-4 Photoproduct; BER: Base Excision Repair; CHX: Cycloheximide; CLUV: Chronic-dose of UV light; CPD: Pyrimidine cyclobutane dimer; DDT: DNA-damage tolerance; DSB: Double-strand break; DUB: Deubiquitylating enzyme; EE: Elementary effect; FACS: Fluorescence activated cell sorter; MMS: Methyl methanesulfonate; NER: Nucleotide Excision Repair; NR: Normalized representation; PCNA: Proliferating Cell Nuclear Antigen; PDB: Protein Data Bank; PRR: Post Replication Repair; PSA: Parameter sweep analysis; SA: Sensitivity analysis; SSA: Stochastic simulation algorithm; ssDNA: Single-stranded DNA; TLS: Translesion DNA Synthesis; TS: Template Switching; UR: Units representation.

## Competing interests

The authors declare that they have no competing interests.

## Authors’ contributions

FA, MMF, DB and PP conceived the study. FA developed the experimental setup and performed the laboratory work. RC defined the model and conducted the computational analyses. DB, PC, DP, FA and RC wrote the manuscript. DB, PC and DP contributed key ideas, supervised the computational work and critically revised the manuscript. MMF and PP contributed key ideas, supervised the laboratory work and assisted in manuscript preparation. ACN critically read the manuscript and provided valuable advices during the progress of the whole work. All authors read and approved the final manuscript.

## Supplementary Material

Additional file 1 Histogram and density plots of CHX effect on protein synthesis and cell cycle progression of UV irradiated cells. Click here for file

Additional file 2 Determination of mono- and poly-ubiquitylated PCNA ratio using western blots elaboration and representation of densitometry quantification. Click here for file

Additional file 3 Global sensitivity analysis of the PRR model. Click here for file

Additional file 4 Graphical representation of reactions ranking obtained by sensitivity analysis. Click here for file

Additional file 5 Methods for the representation of simulation outcomes and comparison with experimental data. Click here for file

Additional file 6 PDB accession codes for protein complexes analyzed through 3D structural modeling. Click here for file

Additional file 7 Structural modeling of Uba1-Rad6 and Rad6-Rad18 complexes involved in PCNA mono-ubiquitylation. Click here for file

Additional file 8 Hypothetical yeast E1-E2 and E2-E3 complexes involved in PCNA mono- and poly-ubiquitylation obtained through structural modeling of PRR complexes. Click here for file

Additional file 9 Structural modeling of Uba1-Ubc13 and Ubc13-Rad5 complexes involved in PCNA poly-ubiquitylation. Click here for file

Additional file 10 **List of reactions involved in the formation of *Ubc13 : Mms2 *complex and simulation results.** Click here for file

Additional file 11 Early time-course of PCNA ubiquitylation after low acute UV irradiation. Click here for file

Additional file 12 Effects of the variation of the initial amount of PCNA. Click here for file

Additional file 13 **Effects of the modulation of the binding between Rad6 and ubiquitin (reaction constant *c***_***4***_**) and of PCNA mono-ubiquitylation (reaction constant *c***_***9***_**).** Click here for file

Additional file 14 **Effects of the modulation of the dissociation of mono-ubiquitylated PCNA from Rad18 dimer (reaction constant *c***_***10***_**) and of the association of Rad5 with mono-ubiquitylated PCNA (reaction constant *c***_***12***_**).** Click here for file

Additional file 15 **Effects of the modulation of the formation of di-ubiquitylated (reaction constant *c***_***16***_**) and of tri-ubiquitylated (reaction constant *c***_***22***_**) PCNA isoforms.** Click here for file

Additional file 16 **FACS analysis of wild type and*rad14δ* background cells irradiated at 10 J/m**^**2**^** UV dose.** Click here for file

Additional file 17 **Deletion of*UBP10* and*UBP15* do not prevent PCNA deubiquitylation at later time-points after low acute UV irradiation.** Click here for file
