# Supplementary material for: In vivo and in silico analysis of PCNA ubiquitylation in the activation of the Post Replication Repair pathway in S. cerevisiae
Source: BMC Syst Biol. 2013 Mar 20;7:24. doi: 10.1186/1752-0509-7-24 (PMC3668150; doi:10.1186/1752-0509-7-24)
Supplement: Additional file 1 — Histogram and density plots of CHX effect on protein synthesis and cell cycle progression of UV irradiated cells. [file 1752-0509-7-24-S1.pdf]

ADDITIONAL FILE 1

Histogram and density plots of CHX effect on protein synthesis and cell cycle progression of UV-irradiated cells

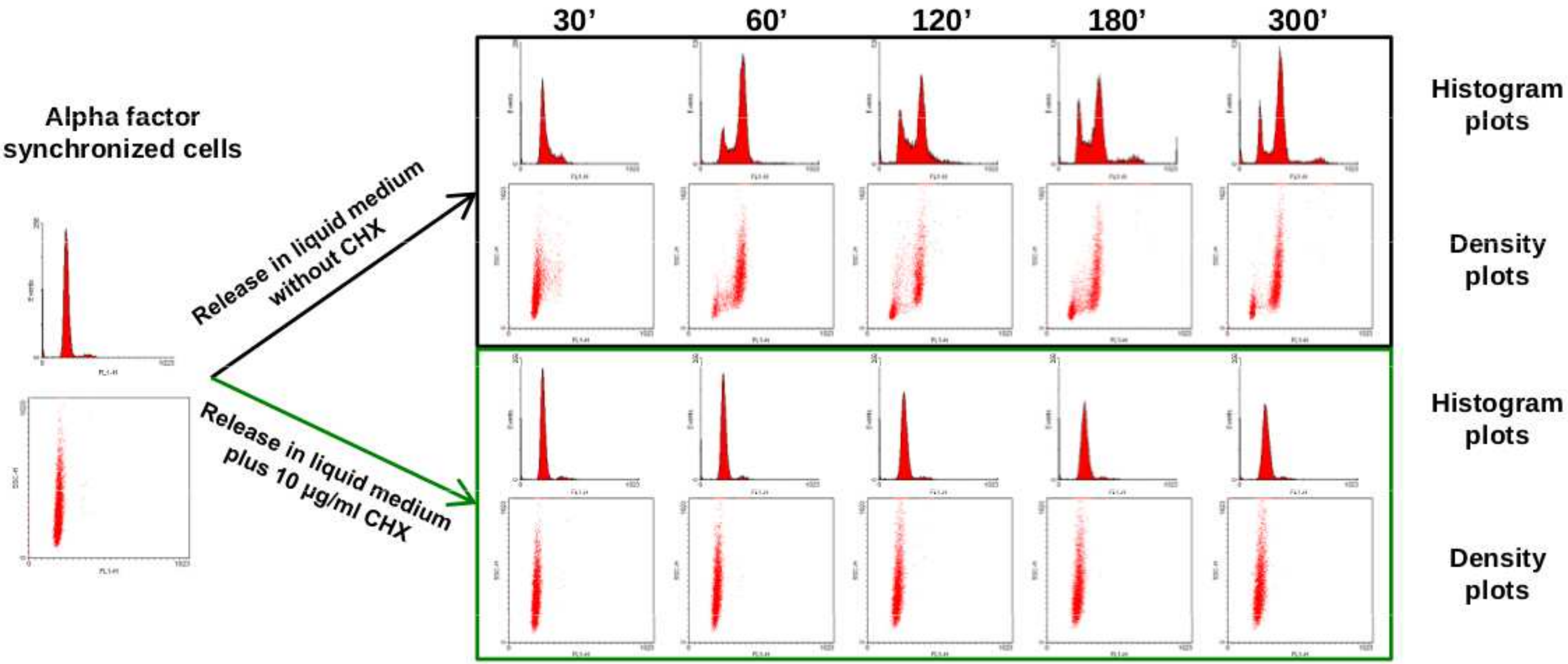

Cells were synchronized through 6 μg/ml of alpha factor and then released in liquid medium without CHX (top graphics) or with 10 μg/ml of CHX (bottom graphics).
In the histogram plots, the left peak corresponds to G1 cells, the right peak corresponds to G2 cells, while S phase cells are in the middle; in the density plots, the left cloud corresponds to G1 cells, the right cloud corresponds to G2 cells, S phase cells are in the middle. Both histogram and density plots at 240 min are not shown, since they are exactly equal to those taken at 180 min and 300 min.
This experiment confirmed that 10 μg/ml of CHX are sufficient to stop cell cycle progression.
