# Supplementary material for: In vivo and in silico analysis of PCNA ubiquitylation in the activation of the Post Replication Repair pathway in S. cerevisiae
Source: BMC Syst Biol. 2013 Mar 20;7:24. doi: 10.1186/1752-0509-7-24 (PMC3668150; doi:10.1186/1752-0509-7-24)
Supplement: Additional file 2 — Determination of mono- and poly-ubiquitylated PCNA ratio using western blots elaboration and representation of densitometry quantification. [file 1752-0509-7-24-S2.pdf]

ADDITIONAL FILE 2

Determination of mono- and poly-ubiquitylated PCNA ratio using western blots: elaboration and representation of densitometry quantification

To quantify the values of mono-/poly-ubiquitylated PCNA ratio from time-course experiments, the ubiquitylation signal of mono- and poly-ubiquitylated PCNA isoforms was normalized with respect to the unmodified PCNA signal obtained on western blots. The whole process was carried out for each UV irradiation dose considered in this work, taking into account all the experiments replicates (3, unless otherwise specified), in order to derive the mean and standard deviation of mono-/poly-ubiquitylated PCNA ratio.

The quantification procedure consisted in the following steps.

1. The initial data are represented by two different types of western blot film for each experiment: "film A", corresponding to the measurement of the unmodified PCNA (see, e.g., bottom part of blot in Figure 2A), and "film B", corresponding to ubiquitylated PCNA (see, e.g., top part of blot in Figure 2A). Seven time measurements $t_1, \ldots, t_7$ – taken at 0, 30, 60, 120, 180, 240, 300 min, respectively – appear on each film. Both films were scanned and quantified by means of the NIH densitometry software ImageJ [1], yielding the initial quantifications $w_1, \ldots, w_7$ of unmodified PCNA (film A), and $w_{1,1}, \ldots, w_{1,7}$, $w_{2,1} \ldots, w_{2,7}$, $w_{3,1}, \ldots, w_{3,7}$ of mono-, di- and tri-ubiquitylated isoforms of PCNA (film B), respectively, for each sampled time point $t_1, \ldots, t_7$.

2. To obtain the correct measurements of unmodified PCNA and of mono-, di- and tri-ubiquitylated PCNA isoforms from western blots, we had to determine the value of the background of each film, which was quantified by using ImageJ, for each UV dose and for each replicate of the experiments. Let us call α and β the background values for film A and B, respectively. Then, for each time point t_i ($i = 1, \ldots, 7$), we processed the previously quantified values of unmodified (w_i) and mono-, di- and tri-ubiquitylated PCNA isoforms ($w_{u,i}$, where $u = 1, 2, 3$) with respect to α and β as follows:

$$w'_i = \begin{cases} w_i - \alpha & \text{if } w_i - \alpha > 0, \\ 0 & \text{otherwise,} \end{cases}$$

   and, for every $u = 1, 2, 3$,

$$w'_{u,i} = \begin{cases} w_{u,i} - \beta & \text{if } w_{u,i} - \beta > 0, \\ 0 & \text{otherwise.} \end{cases}$$

3. The measurements derived at Step 2 for film B need to be normalized with respect to the measurements of the corresponding film A, for each UV dose and for each replicate. To this aim, we first evaluated the normalization coefficient γ_i for each time point t_i ($i = 1, \ldots, 7$) of film A:

$$\gamma_i = \frac{w'_i}{\max_{i=1,\ldots,7}\{w'_i\}},$$

   and then, for each time point t_i and for every $u = 1, 2, 3$, we derived the normalized values of mono-, di- and tri-ubiquitylated isoforms of PCNA on film B with respect to the corresponding values of unmodified PCNA on film A (namely, the value γ_i):

$$w''_{u,i} = \frac{w'_{u,i}}{\gamma_i}.$$

4. Finally, for each western blot film we computed the percentage of the mutual signal intensity of each isoform of ubiquitylated PCNA with respect to the total amount of ubiquitylated PCNA measured on the same film. By taking into account the normalized values of the western blot of all replicates of the experiments, we then evaluated the mean and standard deviation of mono-, di- and tri-ubiquitylated isoforms of PCNA – denoted by $\mu(\div\mathrm{PCNA}^{\mathrm{Ub_u}}_{exp})$ and $\sigma(\div\mathrm{PCNA}^{\mathrm{Ub_u}}_{exp})$, respectively – that were finally used to compare the results of the computational analysis with the experimental data.

[1] Abramoff M, Magelhaes P, Ram S: **Image processing with ImageJ**. *Biophotonics International* 2004, 11(7):36–42.
