# Supplementary material for: In vivo and in silico analysis of PCNA ubiquitylation in the activation of the Post Replication Repair pathway in S. cerevisiae
Source: BMC Syst Biol. 2013 Mar 20;7:24. doi: 10.1186/1752-0509-7-24 (PMC3668150; doi:10.1186/1752-0509-7-24)
Supplement: Additional file 3 — Global sensitivity analysis of the PRR model. [file 1752-0509-7-24-S3.pdf]

ADDITIONAL FILE 3
Global sensitivity analysis of the PRR model

In the mathematical modeling of biological systems, sensitivity analysis (SA) techniques can help in understanding how much the uncertainty in the model outcome is determined by the variation of the model input factors (molecular species amounts, kinetic constants, etc.), besides revealing which input factors bring about the most striking effects on the system behavior, and thus can be assumed to be good control points for its dynamics.

In this work we apply a SA screening test, called "method of the elementary effects" (EE) [1]; in particular, we refer to the improved and refined version of this method presented in [2,3]. The EE method allows to investigate how the model outcome changes according to a perturbation of its input factors, that is realized by varying one factor at a time while keeping all the others fixed. As a result, the EE method associates to each input factor its so-called *elementary effect*, defined as the ratio between the variation in the model output and the variation in the input factor itself. Different elementary effects for each factor are estimated and averaged, in order to compute two global sensitivity measures: the value μ^*, which assesses the overall influence of each factor on the model output, and the value σ^*, which estimates the ensemble of the factor's higher order effects (we refer to [2] for a detailed description of the method).

Concerning the SA of the PRR model, we computed the sensitivity measures μ^* and σ^* by considering as input factors the set of kinetic constants associated to the model reactions. The outcomes of the model used to compute the EEs correspond to the molecular amounts of mono- and poly-ubiquitylated PCNA isoforms, uniformly measured every 9 sec along *in silico* simulations of the dynamics of PRR over 5 hours, considering the 10 J/m^2 UV dose.

The variation interval of the input factors considered in this SA was fixed over 4 orders of magnitude, 2 below and 2 above the reference value of each reaction constant (see Table 3 in the paper). To calculate the sensitivity measures, the set of points of the parameter space used to compute the EE was determined by sampling the variation interval of each reaction constants using the strategy presented in [3], where the Sobol's quasi random numbers [4] were exploited to obtain a radial sampling (log-scaled over the variation interval). Namely, we sampled a set of a_i points, $i = 1, \ldots, 1000$, corresponding to the centers of the radial samplings, and for each a_i we considered a variation along the 25 dimensions of the input factor space to compute the EEs (one for each reaction constant), yielding a total of 1000·(25+1) different model parameterizations.

In Table 1 we show the values of μ^* and σ^* of all reaction constants for the two model outputs – mono-ubiquitylation (left) and poly-ubiquitylation of PCNA (right) – and the ranking of reactions according to decreasing values of μ^*. The horizontal blocks in the tables identify the sets of reactions whose values of μ^* belong to the same order of magnitude. A graphical representation of model reactions ranking is also given in Additional File 4.

Table 1: Ranking of model reactions according to μ^* for the mono-ubiquitylation of PCNA (*left*) and poly-ubiquitylation of PCNA (*right*).

Reaction	μ^*	σ^*
1	1.2146	2.1099
4	$9.6114 \cdot 10^{-2}$	$2.2428 \cdot 10^{-1}$
12	$1.3929 \cdot 10^{-3}$	$1.1950 \cdot 10^{-2}$
18	$8.9356 \cdot 10^{-6}$	$1.1310 \cdot 10^{-4}$
23	$4.6616 \cdot 10^{-6}$	$1.6221 \cdot 10^{-5}$
9	$2.9883 \cdot 10^{-6}$	$9.2425 \cdot 10^{-6}$
25	$2.9548 \cdot 10^{-6}$	$1.4706 \cdot 10^{-5}$
17	$1.7377 \cdot 10^{-6}$	$1.5368 \cdot 10^{-5}$
20	$1.3714 \cdot 10^{-6}$	$8.2233 \cdot 10^{-6}$
21	$8.7619 \cdot 10^{-7}$	$4.4432 \cdot 10^{-6}$
22	$4.3180 \cdot 10^{-7}$	$2.9323 \cdot 10^{-6}$
2	$2.2217 \cdot 10^{-7}$	$1.8861 \cdot 10^{-6}$
7	$7.3924 \cdot 10^{-8}$	$5.0348 \cdot 10^{-7}$
13	$3.6523 \cdot 10^{-8}$	$5.4385 \cdot 10^{-7}$
8	$2.2538 \cdot 10^{-8}$	$1.2463 \cdot 10^{-7}$
15	$2.1505 \cdot 10^{-8}$	$4.7824 \cdot 10^{-7}$
24	$1.1074 \cdot 10^{-8}$	$1.2645 \cdot 10^{-7}$
14	$6.0435 \cdot 10^{-9}$	$4.8142 \cdot 10^{-8}$
19	$5.2410 \cdot 10^{-9}$	$3.4058 \cdot 10^{-8}$
10	$4.9045 \cdot 10^{-10}$	$4.4654 \cdot 10^{-9}$
16	$1.0037 \cdot 10^{-11}$	$2.0655 \cdot 10^{-10}$
3	$2.8808 \cdot 10^{-13}$	$3.5596 \cdot 10^{-12}$
6	$2.4153 \cdot 10^{-13}$	$2.4214 \cdot 10^{-12}$
5	$1.3459 \cdot 10^{-14}$	$1.2814 \cdot 10^{-13}$
11	$6.5943 \cdot 10^{-23}$	$1.7684 \cdot 10^{-21}$

Reaction	μ^*	σ^*
1	$2.4371 \cdot 10^{-1}$	$6.6924 \cdot 10^{-1}$
4	$2.0837 \cdot 10^{-2}$	$7.3272 \cdot 10^{-2}$
12	$5.9092 \cdot 10^{-3}$	$3.1596 \cdot 10^{-2}$
23	$3.5319 \cdot 10^{-5}$	$1.0420 \cdot 10^{-4}$
18	$3.9620 \cdot 10^{-6}$	$5.7120 \cdot 10^{-5}$
21	$2.3807 \cdot 10^{-6}$	$1.2263 \cdot 10^{-5}$
20	$1.5264 \cdot 10^{-6}$	$8.7254 \cdot 10^{-6}$
17	$1.0145 \cdot 10^{-6}$	$8.4656 \cdot 10^{-6}$
9	$6.6420 \cdot 10^{-7}$	$3.1911 \cdot 10^{-6}$
22	$3.0728 \cdot 10^{-7}$	$2.3997 \cdot 10^{-6}$
2	$6.5226 \cdot 10^{-8}$	$7.9826 \cdot 10^{-7}$
13	$4.0320 \cdot 10^{-8}$	$5.4867 \cdot 10^{-7}$
7	$1.7669 \cdot 10^{-8}$	$1.3968 \cdot 10^{-7}$
14	$1.5766 \cdot 10^{-8}$	$1.1217 \cdot 10^{-7}$
15	$1.2015 \cdot 10^{-8}$	$2.1648 \cdot 10^{-7}$
25	$1.0743 \cdot 10^{-8}$	$1.0942 \cdot 10^{-7}$
8	$8.3652 \cdot 10^{-9}$	$6.7295 \cdot 10^{-8}$
19	$5.4418 \cdot 10^{-9}$	$3.7516 \cdot 10^{-8}$
24	$4.8583 \cdot 10^{-9}$	$5.4275 \cdot 10^{-8}$
10	$4.8308 \cdot 10^{-10}$	$3.7435 \cdot 10^{-9}$
16	$1.6753 \cdot 10^{-11}$	$1.5816 \cdot 10^{-10}$
3	$1.4687 \cdot 10^{-13}$	$3.0104 \cdot 10^{-12}$
6	$7.6793 \cdot 10^{-14}$	$7.5556 \cdot 10^{-13}$
5	$4.3665 \cdot 10^{-15}$	$6.0235 \cdot 10^{-14}$
11	$8.1343 \cdot 10^{-23}$	$1.9211 \cdot 10^{-21}$

In order to reduce the huge computational time required to perform this high number of stochastic simulations (around $2.6 \cdot 10^8$, assuming 10^4 independent simulations for each sample to calculate the required histogram distance [5]) that would be needed to carry out a SA on the stochastic model of PRR, we executed here the SA on the deterministic version of the model, derived from the reactions (given in Table 3 of the paper) according to the methodology described in [6,7], and then simulated using the LSODA algorithm [8]. The development and implementation of a SA approach for stochastic models accelerated on Graphics Processing Units is currently in progress, this will allow us to efficiently perform in a parallel fashion all the stochastic simulations required to compute the sensitivity measures of large mechanistic models.

[1] Morris M: **Factorial sampling plans for preliminary computational experiments**. *Technometrics* 1991, **33**(2):161–174.
[2] Campolongo F, Cariboni J, Saltelli A: **An effective screening design for sensitivity analysis of large models**. *Environ Modell Softw* 2007, **22**(10):1509–1518.
[3] Campolongo F, Saltelli A, Cariboni J: **From screening to quantitative sensitivity analysis. A unified approach**. *Comput Phys Commun* 2011, **182**(4):978–988.
[4] Sobol' I: **On the distribution of points in a cube and the approximate evaluation of integrals**. *Zhurnal Vychislitel'noi Matematiki i Matematicheskoi Fiziki* 1967, **7**(4):784–802.
[5] Degasperi A, Gilmore S: **Sensitivity analysis of stochastic models of bistable biochemical reactions**. In *Formal Methods for Computational Systems Biology (SFM-08:Bio)*, LNCS 5016. Edited by Bernardo M, Degano P, Zavattaro G, Berlin, Heidelberg 2008:1–20.
[6] Gillespie DT: **Exact stochastic simulation of coupled chemical reactions**. *J Phys Chem* 1977, **81**(25):2340–2361.
[7] Wolkenhauer O, Ullah M, Kolch W, Kwang-Hyun C: **Modeling and simulation of intracellular dynamics: choosing an appropriate framework**. *IEEE T. Nanobiosci.* 2004, **3**(3):200–7.
[8] Petzold L: **Automatic selection of methods for solving stiff and nonstiff systems of ordinary differential equations**. *SIAM J Sci Stat Comp* 1983, **4**:136–148.
