# Supplementary material for: In vivo and in silico analysis of PCNA ubiquitylation in the activation of the Post Replication Repair pathway in S. cerevisiae
Source: BMC Syst Biol. 2013 Mar 20;7:24. doi: 10.1186/1752-0509-7-24 (PMC3668150; doi:10.1186/1752-0509-7-24)
Supplement: Additional file 4 — Graphical representation of reactions ranking obtained by sensitivity analysis. [file 1752-0509-7-24-S4.pdf]

# ADDITIONAL FILE 4

## Graphical representation of reactions ranking obtained by sensitivity analysis

The graphics below show the values of the sensitivity measure $\mu^*$ of all reaction constants for the two model outputs – mono-ubiquitylation (*top plot*) and poly-ubiquitylation (*bottom plot*) of PCNA – and the ranking of reactions according to decreasing values of $\mu^*$ (see also the tables in Additional File 3). In each plot, the inset represents the ranking on a log-scale.

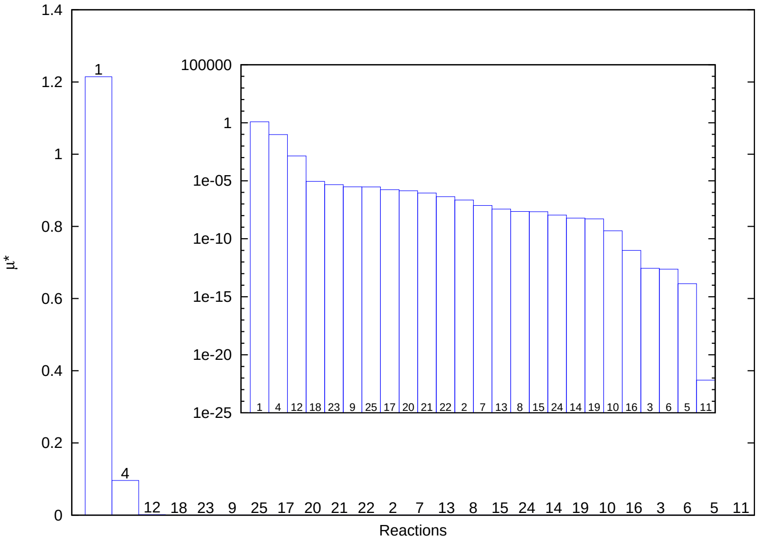


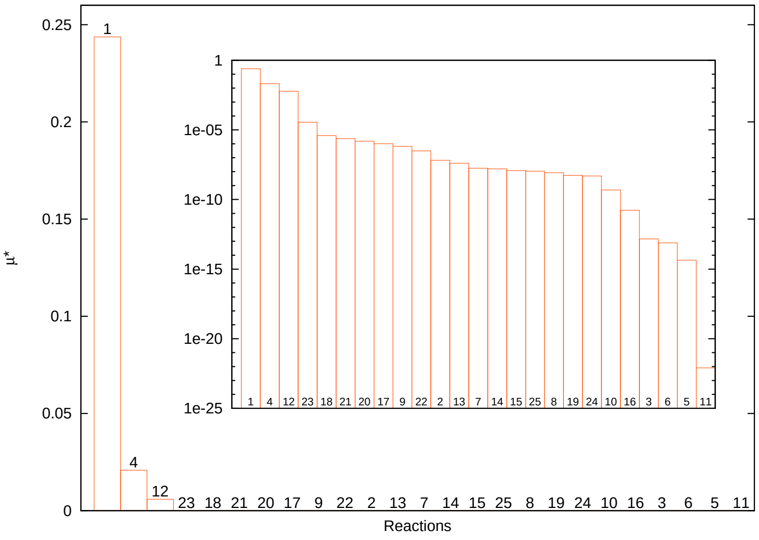


According to the variation of the model input factors considered in this work, the two plots show that, in relation to both model outputs considered for the global sensitivity analysis of the PRR model, the most sensitive reactions (or, in analogous terms, their associated rate constants) are:

- reaction 1, which corresponds to the identification of the UV-induced lesion on DNA. The high global sensitivity of this reaction is motivated by its role in the activation of the whole pathway;
- reaction 4, which corresponds to the loading of ubiquitin on Rad6. This is a fundamental step for PCNA mono-ubiquitylation and, due to the stepwise mechanism of ubiquitylation considered in the model, it also influences the downstream binding of additional moieties to PCNA (i.e., PCNA di-ubiquitylation and tri-ubiquitylation) and, therefore, it affects the whole process of PCNA ubiquitylation;
- reaction 12, which corresponds to the loading of ubiquitin on Rad5. This is a fundamental step for PCNA di-ubiquitylation and tri-ubiquitylation, which is less sensitive in the mono-ubiquitylation output than in the mono-ubiquitylation output since its role is downstream the first steps of the PRR pathway.

All other reactions have a marginal influence on the two model outcomes.
