# Supplementary material for: In vivo and in silico analysis of PCNA ubiquitylation in the activation of the Post Replication Repair pathway in S. cerevisiae
Source: BMC Syst Biol. 2013 Mar 20;7:24. doi: 10.1186/1752-0509-7-24 (PMC3668150; doi:10.1186/1752-0509-7-24)
Supplement: Additional file 5 — Methods for the representation of simulation outcomes and comparison with experimental data. [file 1752-0509-7-24-S5.pdf]

# ADDITIONAL FILE 5

## Methods for the representation of simulation outcomes and comparison with experimental data

For the validation of the PRR model, we compared the outcome of stochastic simulations with the experimental measurements. Since we had to compare different kinds of data – namely, *ratios* of modified PCNA derived from laboratory experiments on the one side, and *molecular amounts* of modified PCNA obtained from stochastic simulations on the other side – we introduced two strategies, called *normalized representation* and *units representation*, for the graphical representation of experimental and computational results.

To this aim, we first quantified the ratio of mono-, di- and tri-ubiquitylated PCNA from western blot experiments for each UV irradiation dose (denoted by $\div\mathrm{PCNA}^{\mathrm{Ub_u}}_{exp}$, where $u = 1, 2, 3$ corresponds to the three ubiquitylated isoforms), as described in Additional File 2. Then, we derived the molecular amounts of mono-, di- and tri-ubiquitylated PCNA (denoted by $\#\mathrm{PCNA}^{\mathrm{Ub_u}}_{sim}$, where $u = 1, 2, 3$ corresponds to the three ubiquitylated isoforms) generated during the stochastic simulations. These values were determined by summing up the molecular amounts of all complexes in which mono-, di- and tri-ubiquitylated PCNA isoforms appeared in the system during the simulations (see Table 3 in the paper). In order to take into account the effects of stochastic fluctuations, a set of independent stochastic simulations were performed to compute the mean $\mu(\#\mathrm{PCNA}^{\mathrm{Ub_u}}_{sim})$ and standard deviation $\sigma(\#\mathrm{PCNA}^{\mathrm{Ub_u}}_{sim})$ of these modified PCNA amounts. Moreover, in both representations the values of di- and tri-ubiquitylated forms of simulated PCNA were represented as the sum of $\#\mathrm{PCNA}^{\mathrm{Ub_1}}_{sim}+\#\mathrm{PCNA}^{\mathrm{Ub_2}}_{sim}$ and $\#\mathrm{PCNA}^{\mathrm{Ub_1}}_{sim}+\#\mathrm{PCNA}^{\mathrm{Ub_2}}_{sim}+\#\mathrm{PCNA}^{\mathrm{Ub_3}}_{sim}$, respectively, to be correctly compared with the corresponding experimental measurements.

1. The *normalized representation* (NR) strategy consists of stacked bar graphs: for each sampled time point $t_1, \dots, t_7$ within the measurement interval 0-5 h, the stacked bars corresponding to the normalized values of the computational outcomes (denoted by $\div\mathrm{PCNA}^{\mathrm{Ub_u}}_{sim}$) are plotted side by side to the experimental bars $\div\mathrm{PCNA}^{\mathrm{Ub_u}}_{exp}$ (which are already expressed as ratios, as described in Additional File 2). With NR, the "normalized stacked bars" corresponding to the computational outcomes were derived as follows: we first run a stochastic simulation of the model and acquired the values of $\#\mathrm{PCNA}^{\mathrm{Ub_u}}_{sim}$ occurring at times points $t_1, \dots, t_7$; then, for each $t_i$, we derived and plotted the three bar portions $\div\mathrm{PCNA}^{\mathrm{Ub_u}}_{sim}$ corresponding to the normalized values of mono-, di- and tri-ubiquitylated PCNA ratios occurring during the simulation. We remark that the NR allows a direct comparison between the experimental and simulation results, by considering the ratio of the three ubiquitylated isoforms of PCNA with respect to the total amount of modified PCNA measured in the system. Nonetheless, this strategy does not give any information related to the molecular amounts of the mono-, di- and tri-ubiquitylated isoforms of PCNA obtained through stochastic simulations.
2. The *units representation* (UR) strategy overcomes the drawback of NR, since it allows to directly compare the outcomes of stochastic simulations with the western blot quantifications which, in this case, are specifically transformed into molecular quantities. To this aim, the experimental measured ratios $\div\mathrm{PCNA}^{\mathrm{Ub_u}}_{exp}$ were converted into molecular amounts (i.e., number of molecules per cell) by exploiting the results of stochastic simulations: by considering the values of $\#\mathrm{PCNA}^{\mathrm{Ub_u}}_{sim}$ obtained at each time instant $t_i$ during a simulation, we computed the value $S = \sum_{u=1,2,3} \#\mathrm{PCNA}^{\mathrm{Ub_u}}_{sim}$ at time $t_i$, $i = 1, \dots, 7$. Then, we evaluated the quantities

$$\begin{aligned}
\#\mathrm{PCNA}^{\mathrm{Ub_1}}_{exp} &= \div\mathrm{PCNA}^{\mathrm{Ub_1}}_{exp} \times S,\\
\#\mathrm{PCNA}^{\mathrm{Ub_2}}_{exp} &= (\div\mathrm{PCNA}^{\mathrm{Ub_1}}_{exp} + \div\mathrm{PCNA}^{\mathrm{Ub_2}}_{exp}) \times S,\\
\#\mathrm{PCNA}^{\mathrm{Ub_3}}_{exp} &= (\div\mathrm{PCNA}^{\mathrm{Ub_1}}_{exp} + \div\mathrm{PCNA}^{\mathrm{Ub_2}}_{exp} + \div\mathrm{PCNA}^{\mathrm{Ub_3}}_{exp}) \times S.
\end{aligned}$$

   The variables $\#\mathrm{PCNA}^{\mathrm{Ub_u}}_{exp}$ represent the experimental measurements transformed from percentages (i.e., ratio of each modified isoform of PCNA with respect to the total amount of modified PCNA experimentally measured) into units (i.e., number of molecules of each modified isoform of PCNA with respect to the total molecular amounts of modified PCNA occurring in the simulation). So doing, the UR of the experimental results can be directly compared to the simulation outcomes. The UR allows to evidence the different dynamics emerging from the system and, in particular, it clearly represents the switch-off of the ubiquitylation signal as long as the DNA lesions get processed. This kind of information is not shown with the NR of the simulated and experimental ratios, since the NR does not give any knowledge on the actual amount of modified PCNA corresponding to the height of the stacked bars.

Finally, we stress the fact that whilst the NR is a direct display of the experimental measurements, the UR is markedly related to the computational outcomes and, as such, it hinges upon the parameterization used to run the stochastic simulations. This means that if we change the parameters (either the initial molecular amounts or the stochastic constants, or both) and run a new simulation, the computational results, and hence also the values $\#\mathrm{PCNA}^{\mathrm{Ub_u}}_{exp}$, vary. Therefore, the UR graphs cannot give a certain knowledge on the actual cellular amounts of mono- and poly-ubiquitylated isoforms of PCNA, but nevertheless it provides useful hints on the system dynamics.

We also remark that additional sources of uncertainty, possibly able to impair the direct comparison between the experimental data and the simulation outcomes, and that can be even more evidenced with both NR and UR representation methods, are due to two factors: on the one hand, the possible discrepancy between the amounts of some key regulatory molecules that are effectively occurring *in vivo* and those considered in simulations; on the other hand, the modeling simplification of complex biochemical processes – taking place through a cascade of multiple reactions – reduced to a single reaction or a set of few reactions. In the PRR model, an example of such simplification is related to the ubiquitin activation step mediated by Uba1, a biochemical process consisting in about 20 biochemical reactions which was modeled as a single reaction (see reaction 4, Table 3, and the discussion in section "The PCNA ubiquitylation model" within the paper). Model simplification is a common and essential practice both for developing models at an appropriate level of abstraction and for reducing the computational costs, notwithstanding the fact that the presence of multiple step processes modeled as single reactions can amplify the discrepancy between the values of the experimental and simulated standard deviations, as previously shown in [1,2].


[1] Pedraza J, Paulsson J: **Effects of molecular memory and bursting on fluctuations in gene expression.** *Science* 2008, 319(5861):339–343.

[2] Csikász-Nagy A, Mura I: **Role of mRNA gestation and senescence in noise reduction during the cell cycle.** *Stud Health Technol Inform* 2011, 162:236–43.
