# Supplementary material for: In vivo and in silico analysis of PCNA ubiquitylation in the activation of the Post Replication Repair pathway in S. cerevisiae
Source: BMC Syst Biol. 2013 Mar 20;7:24. doi: 10.1186/1752-0509-7-24 (PMC3668150; doi:10.1186/1752-0509-7-24)
Supplement: Additional file 6 — PDB accession codes for protein complexes analyzed through 3D structural modeling. [file 1752-0509-7-24-S6.pdf]

ADDITIONAL FILE 6
PDB accession codes for protein complexes analyzed through 3D structural modeling

PDB accession codes for proteins and protein complexes analyzed through the structural modeling approach (see also Additional Files 7 and 9).

**Protein/complex**	**PDB accession code**
UBA3-Ubc12	1Y8X
Uba1	3CMM
Rad6	1AYZ
UbcH7-c-CBL(RING)	1FVB
Ubc13-Mms2	2GMI
