# Supplementary material for: In vivo and in silico analysis of PCNA ubiquitylation in the activation of the Post Replication Repair pathway in S. cerevisiae
Source: BMC Syst Biol. 2013 Mar 20;7:24. doi: 10.1186/1752-0509-7-24 (PMC3668150; doi:10.1186/1752-0509-7-24)
Supplement: Additional file 7 — Structural modeling of Uba1-Rad6 and Rad6-Rad18 complexes involved in PCNA mono-ubiquitylation. [file 1752-0509-7-24-S7.pdf]

ADDITIONAL FILE 7

Structural modeling of Uba1-Rad6 and Rad6-Rad18 complexes involved in PCNA mono-ubiquitylation

Following the procedure published in [1], we constructed the hypothetical yeast E1-E2 complex Uba1-Rad6 (Figure A, Additional File 8) by using as template the crystallized complex UBA3(UFD)-Ubc12(core) formed by the catalytic subunit of the human E1 UBA3-NAE1 and the human E2 Ubc12. These two proteins share high structural conservation with yeast Uba1 (UFD domain) and Rad6, respectively [1]. Then, we superimposed the published 3D structures of yeast Uba1 and Rad6 on the UBA3(UFD)-Ubc12(core) template, and we finally deleted the human proteins from the workbench, obtaining the hypothetical Uba1-Rad6 yeast complex. This complex was analyzed by Protein Interfaces, Surfaces and Assemblies (PISA) server [2] to identify the residues through which Rad6 interacts with Uba1. We determined that 9 residues of Rad6 (Met10, Arg11, Asp12, Phe13, Lys14, Arg15, Met16, Lys17, Glu18 of N-term alpha helix H1), which resulted to be involved in the interaction with Uba1, were experimentally found to be involved also in the interaction with Rad18 [3]. This result suggests the mutual exclusivity of the two yeast complexes Uba1-Rad6 and Rad6-Rad18.

We further confirmed this prediction by constructing the hypothetical yeast complex Rad6-Rad18(RING). For this purpose we used the human E2 UbcH7-c-CBL(RING) complex as 3D template, which is the crystallized complex between the human E2 UbcH7 and the RING domain of human c-CBL. We superimposed the yeast Rad6 structure on this template and, after deleting the UbcH7 structure from the virtual workbench, we started the threading task. Based on the sequence alignment between yeast Rad18(RING) and human c-CBL(RING), we manually threaded the yeast Rad18(RING) onto the fold of human c-CBL(RING), which was then deleted from the virtual workbench. The resulting hypothetical structure of yeast Rad6-Rad18(RING) complex (Figure B, Additional File 8) was analyzed by PISA server, obtaining the putative residues of yeast Rad6 interacting with yeast Rad18. Our predictions were then compared with previous experimental data [3], which confirmed that we correctly predicted 6 residues of the yeast Rad6 (Met10, Arg11, Asp12, Phe13, Lys14, Lys17 of N-term alpha helix H1) interacting with Rad18(RING).

The structural modeling analysis carried out on these two hypothetical yeast complexes E1-E2 Uba1-Rad6 and E2-E3 Rad6-Rad18 allowed us to deduce that the two complexes are mutually exclusive, and that the mechanism of PCNA mono-ubiquitylation, from ubiquitin activation to its covalent linkage on PCNA, is a step-wise process, as previously suggested in [4, 5].

[1] Lee I, Schindelin H: **Structural insights into E1-catalyzed ubiquitin activation and transfer to conjugating enzymes.** *Cell* 2008, **134**(2):268–278.

[2] Krissinel E, Henrick K: **Inference of macromolecular assemblies from crystalline state**. *J Mol Biol* 2007, **372**(3):774–797.

[3] Bailly V, Prakash S, Prakash L: **Domains required for dimerization of yeast Rad6 ubiquitin-conjugating enzyme and Rad18 DNA binding protein**. *Mol Cell Biol* 1997, **17**(8):4536–4543.

[4] Eletr ZM, Huang DT, Duda DM, Schulman BA, Kuhlman B: **E2 conjugating enzymes must disengage from their E1 enzymes before E3-dependent ubiquitin and ubiquitin-like transfer**. *Nat Struct Mol Biol* 2005, **12**(10):933–934.

[5] Parker J, Ulrich HD: **Mechanistic analysis of PCNA poly-ubiquitylation by the ubiquitin protein ligases Rad18 and Rad5**. *EMBO J* 2009, **28**:3657–3666.
