# Supplementary material for: In vivo and in silico analysis of PCNA ubiquitylation in the activation of the Post Replication Repair pathway in S. cerevisiae
Source: BMC Syst Biol. 2013 Mar 20;7:24. doi: 10.1186/1752-0509-7-24 (PMC3668150; doi:10.1186/1752-0509-7-24)
Supplement: Additional file 8 — Hypothetical yeast E1-E2 and E2-E3 complexes involved in PCNA mono- and poly-ubiquitylation obtained through structural modeling of PRR complexes. [file 1752-0509-7-24-S8.pdf]

ADDITIONAL FILE 8

Hypothetical yeast E1-E2 and E2-E3 complexes involved in PCNA mono- and poly-ubiquitylation obtained through structural modeling of PRR complexes

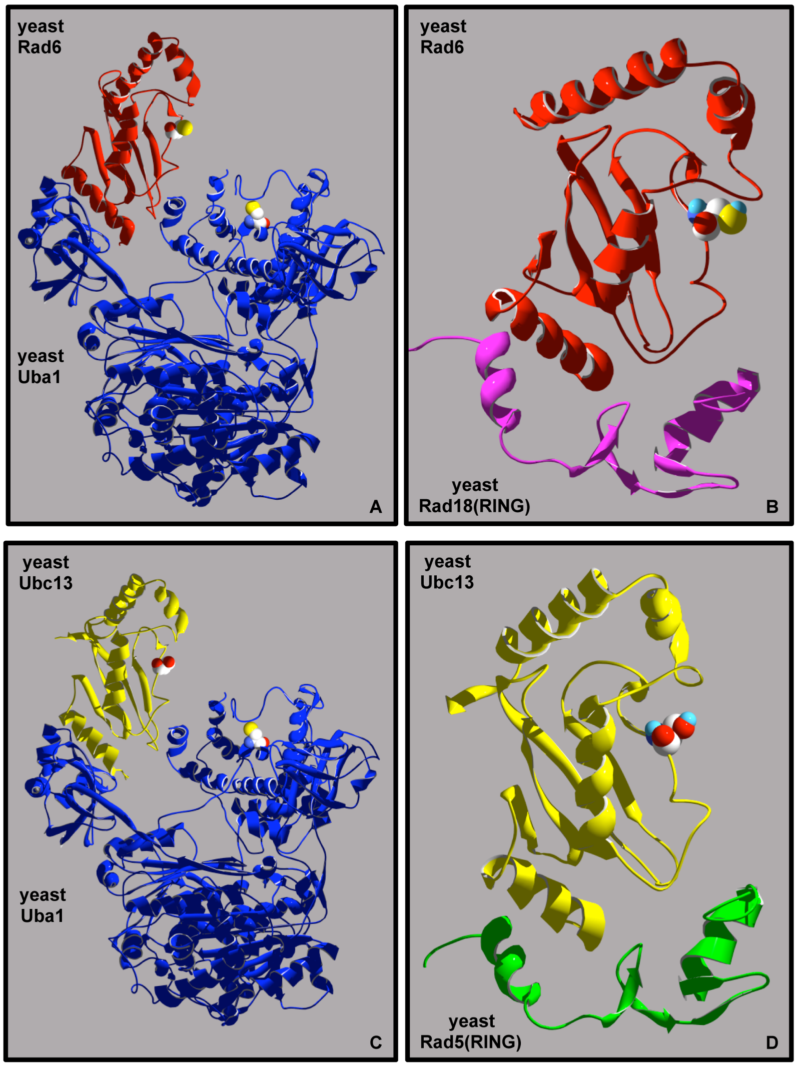

(A) Hypothetical yeast E1-E2 complex Uba1(blue)-Rad6(red).
(B) Hypothetical yeast E2-E3(RING) complex Rad6(red)-Rad18(RING)(magenta).
(C) Hypothetical yeast E1-E2 complex Uba1(blue)-Ubc13(yellow).
(D) Hypothetical yeast E2-E3(RING) complex Ubc13(yellow)-Rad5(RING)(green).

The active sites of the proteins are rendered in solid 3D, namely, Cys600 for Uba1, Cys88 for Rad6, Cys87 for Ubc13.
