# Supplementary material for: In vivo and in silico analysis of PCNA ubiquitylation in the activation of the Post Replication Repair pathway in S. cerevisiae
Source: BMC Syst Biol. 2013 Mar 20;7:24. doi: 10.1186/1752-0509-7-24 (PMC3668150; doi:10.1186/1752-0509-7-24)
Supplement: Additional file 9 — Structural modeling of Uba1-Ubc13 and Ubc13-Rad5 complexes involved in PCNA poly-ubiquitylation. [file 1752-0509-7-24-S9.pdf]

ADDITIONAL FILE 9
Structural modeling of Uba1-Ubc13 and Ubc13-Rad5 complexes involved in PCNA poly-ubiquitylation

Following the procedure published in [1], we constructed the hypothetical yeast Uba1-Ubc13 and Uba1-Ubc13-Mms2 complexes using the template UBA3(UFD)-Ubc12(core), in combination with the Uba1 structure and the published crystallographic structure of yeast Ubc13-Mms2 complex.
We constructed and analyzed the hypothetical yeast Uba1-Ubc13 complex (Figure C, Additional File 8) through the Protein Interfaces, Surfaces and Assemblies (PISA) server [2], identifying the Ubc13 residues involved in the interaction with Uba1. At least two residues of Ubc13 (Lys6 and Lys10 of N-term alpha helix H1), which interact with Uba1, were previously found to be necessary also for the interaction with the E3 enzyme Rad5 [3]. This result suggests the mutual exclusivity between the two complexes Uba1-Ubc13 and Ubc13-Rad5. Moreover, from the analysis of the hypothetical complex Uba1-Ubc13-Mms2, we determined that there are unrealistic contacts between Uba1 and Mms2, deducing that also Uba1-Ubc13 and Ubc13-Mms2 are mutually exclusive: Ubc13 needs to be charged with ubiquitin by Uba1 before binding Mms2.
We confirmed the mutual exclusivity of Uba1-Ubc13 and Ubc13-Rad5 by constructing the hypothetical yeast complex Ubc13-Rad5(RING), exploiting the same approach described in Additional File 7. We used the human E2 UbcH7-c-CBL(RING) complex as main template (this was previously used also by H.D. Ulrich to design mutations on the putative surface of Ubc13-Rad5 [3]). On this template we first superimposed the structure of yeast Ubc13 and then deleted UbcH7 from the virtual workbench; based on the sequence alignment between human c-CBL(RING) and yeast Rad5(RING), we manually threaded the primary sequence of yeast Rad5 onto the fold of human c-CBL(RING). The resulting structure (Figure D, Additional File 8) was analyzed by PISA server. Through this analysis, we assessed that our predictions are in perfect agreement with previously published experimental data [3], confirming that the yeast Uba1-Ubc13 and Ubc13-Rad5 complexes are mutually exclusive.
Our deductions are in good agreement with all published data, which support a distributive/step-wise sequence of events for PCNA poly-ubiquitylation, from ubiquitin activation to its covalent linkage on mono-ubiquitylated PCNA [4, 5].

[1] Lee I, Schindelin H: **Structural insights into E1-catalyzed ubiquitin activation and transfer to conjugating enzymes.** *Cell* 2008, **134**(2):268–278.

[2] Krissinel E, Henrick K: **Inference of macromolecular assemblies from crystalline state**. *J Mol Biol* 2007, **372**(3):774–797.

[3] Ulrich HD: **Protein-protein interactions within an E2-RING finger complex**. *J Biol Chem* 2003, **278**(9):7051–7058.

[4] Eletr ZM, Huang DT, Duda DM, Schulman BA, Kuhlman B: **E2 conjugating enzymes must disengage from their E1 enzymes before E3-dependent ubiquitin and ubiquitin-like transfer**. *Nat Struct Mol Biol* 2005, **12**(10):933–934.

[5] Parker J, Ulrich HD: **Mechanistic analysis of PCNA poly-ubiquitylation by the ubiquitin protein ligases Rad18 and Rad5**. *EMBO J* 2009, **28**:3657–3666.
