# Supplementary material for: In vivo and in silico analysis of PCNA ubiquitylation in the activation of the Post Replication Repair pathway in S. cerevisiae
Source: BMC Syst Biol. 2013 Mar 20;7:24. doi: 10.1186/1752-0509-7-24 (PMC3668150; doi:10.1186/1752-0509-7-24)
Supplement: Additional file 10 — List of reactions involved in the formation of Ubc13 : Mms2 complex and simulation results. [file 1752-0509-7-24-S10.pdf]

ADDITIONAL FILE 10

List of reactions involved in the formation of $Ubc13{:}Mms2$ complex and simulation results

Table 1 reports the list of biochemical reactions involved in the formation of $Ubc13{:}Mms2$ complex, which were removed from an initial version of the model.

Figure 1 shows the average dynamics of mono-ubiquitylated PCNA (blue line) and poly-ubiquitylated PCNA (red line) at 5 J/m^2 UV dose, obtained with (dashed lines) and without (solid lines) the set of reactions given in Table 1. In both cases, the average dynamics were evaluated by considering 100 independent stochastic simulations, executed starting from the same initial conditions and with an estimated number of DNA lesions equal to 1001. The initial conditions for dashed line dynamics are given in Table 1 hereafter, together with Tables 2 and 3 in the paper, while for solid line dynamics they are reported in Tables 2 and 3 in the paper.

Table 1: Removed reactions

Reaction	Reagents	Products	Constant [sec^{-1}]	Reference
1	$Ubc13 + Mms2$	$Ubc13{:}Mms2$	1.82×10^{-1}	[1–4]
2	$Ubc13{:}Mms2$	$Ubc13 + Mms2$	98	[1–4]
3	$Ubc13{:}U{:}Mms2$	$Ubc13{:}Mms2 + U$	1.82×10^{-1}	[1–4]

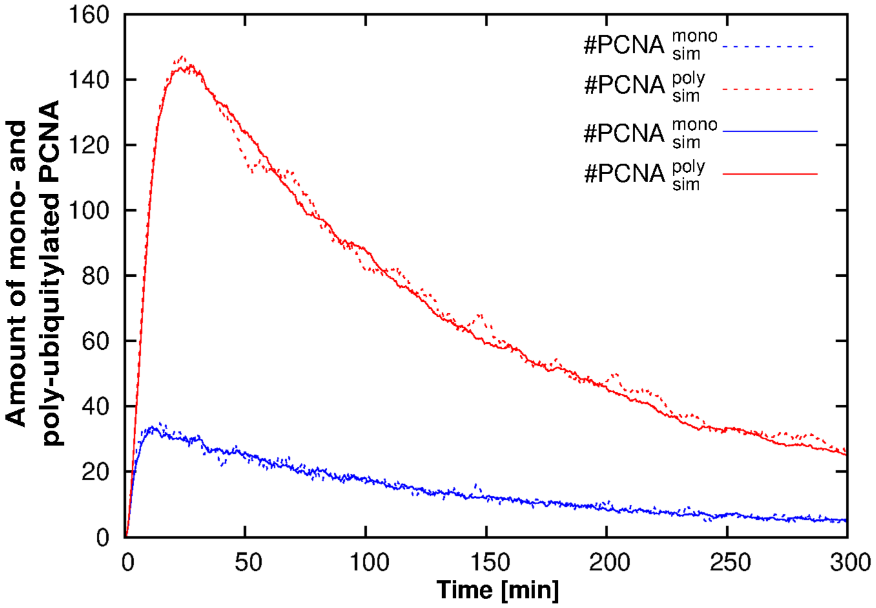

Figure 1: Comparison between the simulated dynamics of PCNA ubiquitylation obtained with (dashed lines) and without (solid lines) the set of reactions listed in Table 1

[1] Hoege C, Pfander B, Moldovan G, Pyrowolakis G, Jentsch S: **RAD6-dependent DNA repair is linked to modification of PCNA by ubiquitin and SUMO**. *Nature* 2002, **419**:135–141.

[2] Parker J, Ulrich HD: **Mechanistic analysis of PCNA poly-ubiquitylation by the ubiquitin protein ligases Rad18 and Rad5**. *EMBO J* 2009, **28**:3657–3666.

[3] Ulrich HD: **Protein-protein interactions within an E2-RING finger complex**. *J Biol Chem* 2003, **278**(9):7051–7058.

[4] Ulrich HD, Jentsch S: **Two RING finger proteins mediate cooperation between ubiquitin-conjugating enzymes in DNA repair**. *EMBO J* 2000, **19**(13):3388–3397.
