# Supplementary material for: In vivo and in silico analysis of PCNA ubiquitylation in the activation of the Post Replication Repair pathway in S. cerevisiae
Source: BMC Syst Biol. 2013 Mar 20;7:24. doi: 10.1186/1752-0509-7-24 (PMC3668150; doi:10.1186/1752-0509-7-24)
Supplement: Additional file 11 — Early time-course of PCNA ubiquitylation after low acute UV irradiation. [file 1752-0509-7-24-S11.pdf]

ADDITIONAL FILE 11

Early time-course of PCNA ubiquitylation after low acute UV irradiation

The figure shows the time-course measurement of mono-, di- and tri-ubiquitylated PCNA isoforms (top part, denoted by α-Ub) and of non modified PCNA (bottom part, denoted by α-His), sampled from 0 to 20 min after UV irradiation.

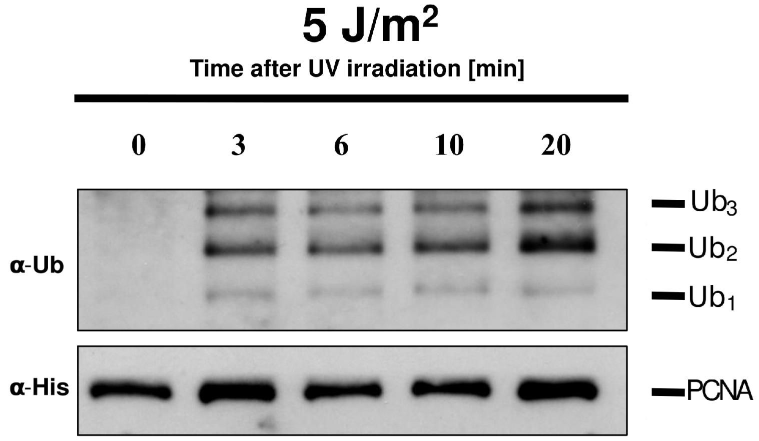

In order to enable the sampling of cells at early time points, we adopted a different protocol of UV irradiation: cells were UV irradiated at 5 J/m^2 and lysed on plate at the indicated time-points without liquid holding (as we instead did for all the experiments of this study). PCNA ubiquitylated isoforms were detected after denaturating pull-down, SDS-urea page and western blotting, as described in Section "Methods".
