# Supplementary material for: In vivo and in silico analysis of PCNA ubiquitylation in the activation of the Post Replication Repair pathway in S. cerevisiae
Source: BMC Syst Biol. 2013 Mar 20;7:24. doi: 10.1186/1752-0509-7-24 (PMC3668150; doi:10.1186/1752-0509-7-24)

# ADDITIONAL FILE 12
## Effects of the variation of the initial amount of PCNA

The figure shows the simulated dynamics of the ubiquitylated PCNA isoforms resulting from a PSA on the amount of PCNA initially present inside the system; simulations were executed assuming an UV dose equal to 10 J/m$^2$. The molecular amount of PCNA was varied in the interval $[0, 14960]$, mimicking the biological effect ranging from the deletion to a 2-fold overexpression of the DNA sliding clamp. The reference dynamics, obtained by using the value of PCNA equal to 7480 molecules, is marked with a red arrow in the plots.
The simulations show that for amounts of PCNA lower than the reference value, the amount of modified PCNA isoforms is strongly reduced and we no longer observe the signal switch-off within 300 min of simulated time. Conversely, by incrementing the amount of PCNA, we observe an increase in the initial peak of all PCNA isoforms and the subsequent signal switch-off.

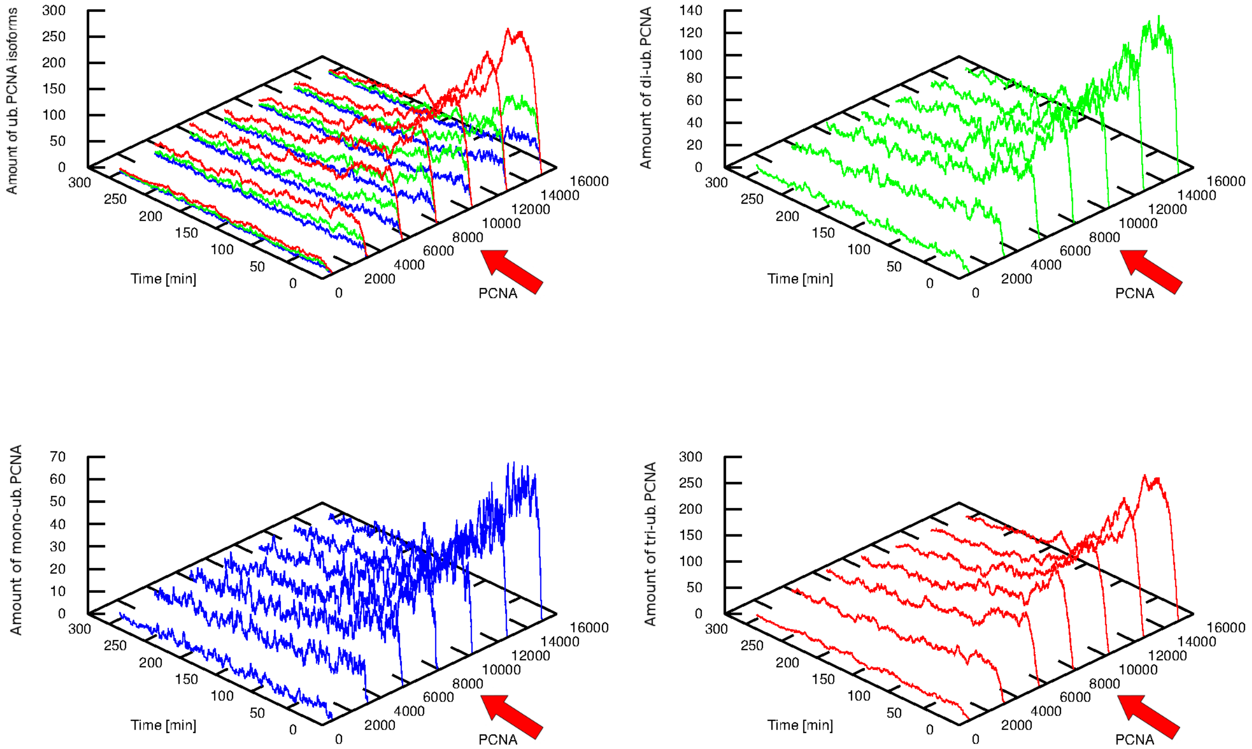

Supplement: Additional file 12 — Effects of the variation of the initial amount of PCNA. [file 1752-0509-7-24-S12.pdf]
