# Supplementary material for: In vivo and in silico analysis of PCNA ubiquitylation in the activation of the Post Replication Repair pathway in S. cerevisiae
Source: BMC Syst Biol. 2013 Mar 20;7:24. doi: 10.1186/1752-0509-7-24 (PMC3668150; doi:10.1186/1752-0509-7-24)

ADDITIONAL FILE 13

Effects of the modulation of the binding between Rad6 and ubiquitin (reaction constant c_4) and of PCNA mono-ubiquitylation (reaction constant c_9)

The figure shows the simulated dynamics of the ubiquitylated PCNA isoforms resulting from two PSA, the first executed on the value of the reaction constant that modulates the binding between Rad6 and ubiquitin (top frame), and the second executed on the reaction constant that rules PCNA mono-ubiquitylation (bottom frame). All simulations were executed assuming an UV dose equal to 10 J/m^2.

The two varied parameters and the corresponding sweep ranges are: (1) constant c_4, within the interval $[2.5 \times 10^{-10}, 2.5 \times 10^{-4}]$; (2) constant c_9, within the interval $[1 \times 10^{-5}, 10]$. The dynamics corresponding to the reference values (see Table 3) are marked with a red box.

These two reactions are involved in the ubiquitin transfer process: reaction 4 loads the ubiquitin on Rad6, while reaction 9 transfers the ubiquitin from Rad6 to PCNA. In both cases, for values of the reaction constant lower than the reference value, we observe low amounts of all modified PCNA isoforms. Conversely, by increasing the value of the constant, that is, by enhancing the binding affinity between Rad6 or ubiquitin and the formation of mono-ubiquitylated PCNA, the obtained dynamics are comparable with the reference one, since we observe the same initial increase in the amount of all PCNA isoforms and the following ubiquitylation signal switch-off.

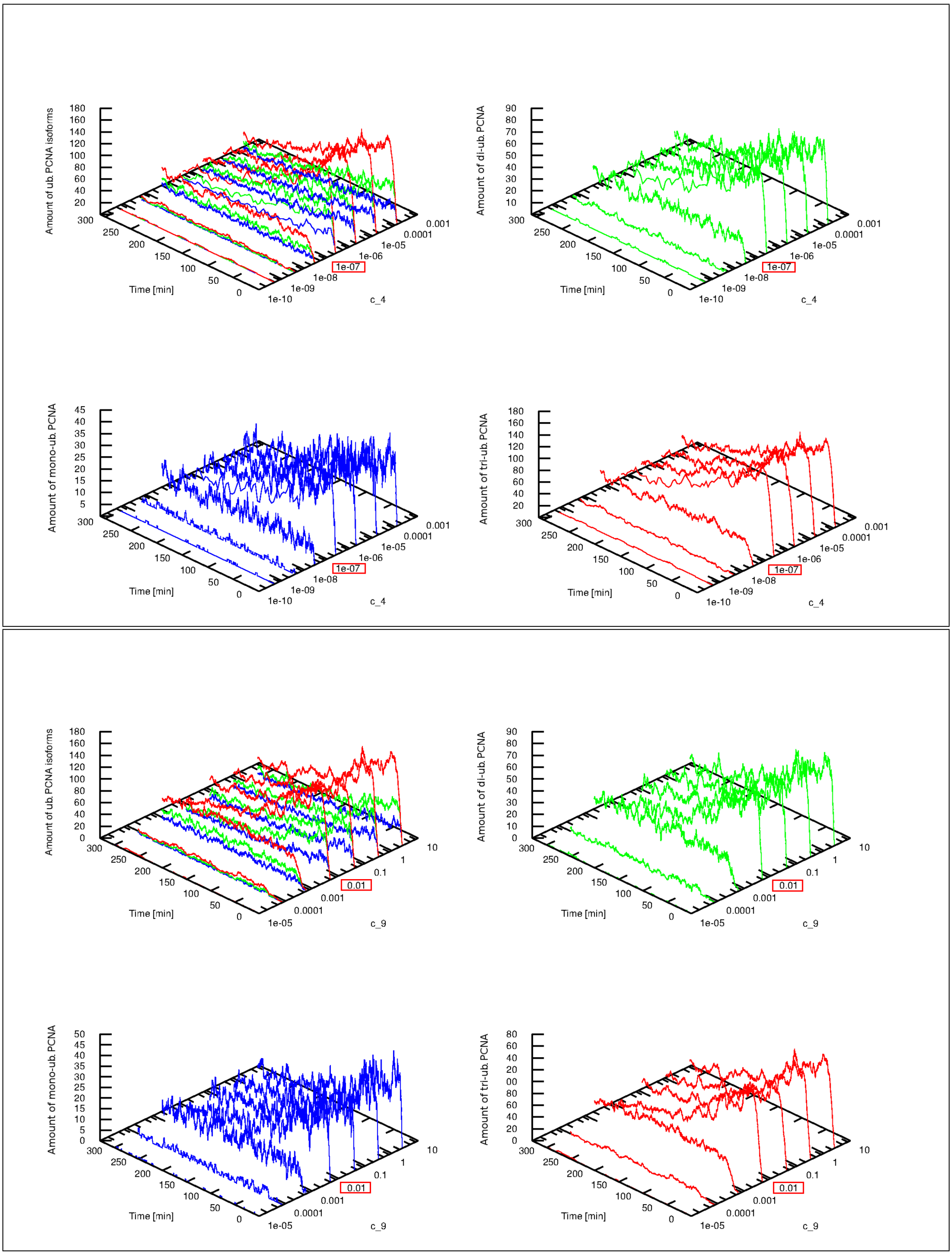

Supplement: Additional file 13 — Effects of the modulation of the binding between Rad6 and ubiquitin (reaction constant c4) and of PCNA mono-ubiquitylation (reaction constant c9). [file 1752-0509-7-24-S13.pdf]
