# Supplementary material for: In vivo and in silico analysis of PCNA ubiquitylation in the activation of the Post Replication Repair pathway in S. cerevisiae
Source: BMC Syst Biol. 2013 Mar 20;7:24. doi: 10.1186/1752-0509-7-24 (PMC3668150; doi:10.1186/1752-0509-7-24)

ADDITIONAL FILE 14

Effects of the modulation of the dissociation of mono-ubiquitylated PCNA from Rad18 dimer (reaction constant c_{10}) and of the association of Rad5 with mono-ubiquitylated PCNA (reaction constant c_{12})

The figure shows the simulated dynamics of the ubiquitylated PCNA isoforms resulting from two PSA, the first executed on the value of the reaction constant that modulates the dissociation of mono-ubiquitylated PCNA from Rad18 dimer (top frame), and the second executed on the association constant of Rad5 with mono-ubiquitylated PCNA (bottom frame). All simulations were executed assuming an UV dose equal to 10 J/m^2.
The two varied parameters and the corresponding sweep ranges are: (1) constant c_{10}, within the interval $[1 \times 10^{-3}, 1 \times 10^{3}]$; (2) constant c_{12}, within the interval $[5 \times 10^{-9}, 5 \times 10^{-3}]$. The dynamics corresponding to the reference values (see Table 3) are marked with a red box.
For values of reaction constant c_{10} lower or equal to 1×10^{-3} we observe an accumulation of the mono-ubiquitylated PCNA isoform, since this constant rules the dissociation activity of mono-ubiquitylated PCNA from Rad18 dimer. Conversely, for higher values of this constant, the resulting dynamics is comparable to the reference one, suggesting that the reaction able to modulate the amount of mono- and poly-ubiquitylated PCNA isoforms is upstream to reaction 10.
Very low values of constant c_{12} (equal or lower than 1×10^{-8}) induce an accumulation of the mono-ubiquitylated PCNA isoform in the system, while keeping the amount of the other PCNA isoforms around zero. This is due to the fact that reaction 12 forms the trimer $Rad5$:$PCNA_{\text{on}}$:U, which is a control point for the switch between PCNA mono- and poly-ubiquitylation. On the other hand, higher values of this constant result in a dynamics comparable to the reference one.

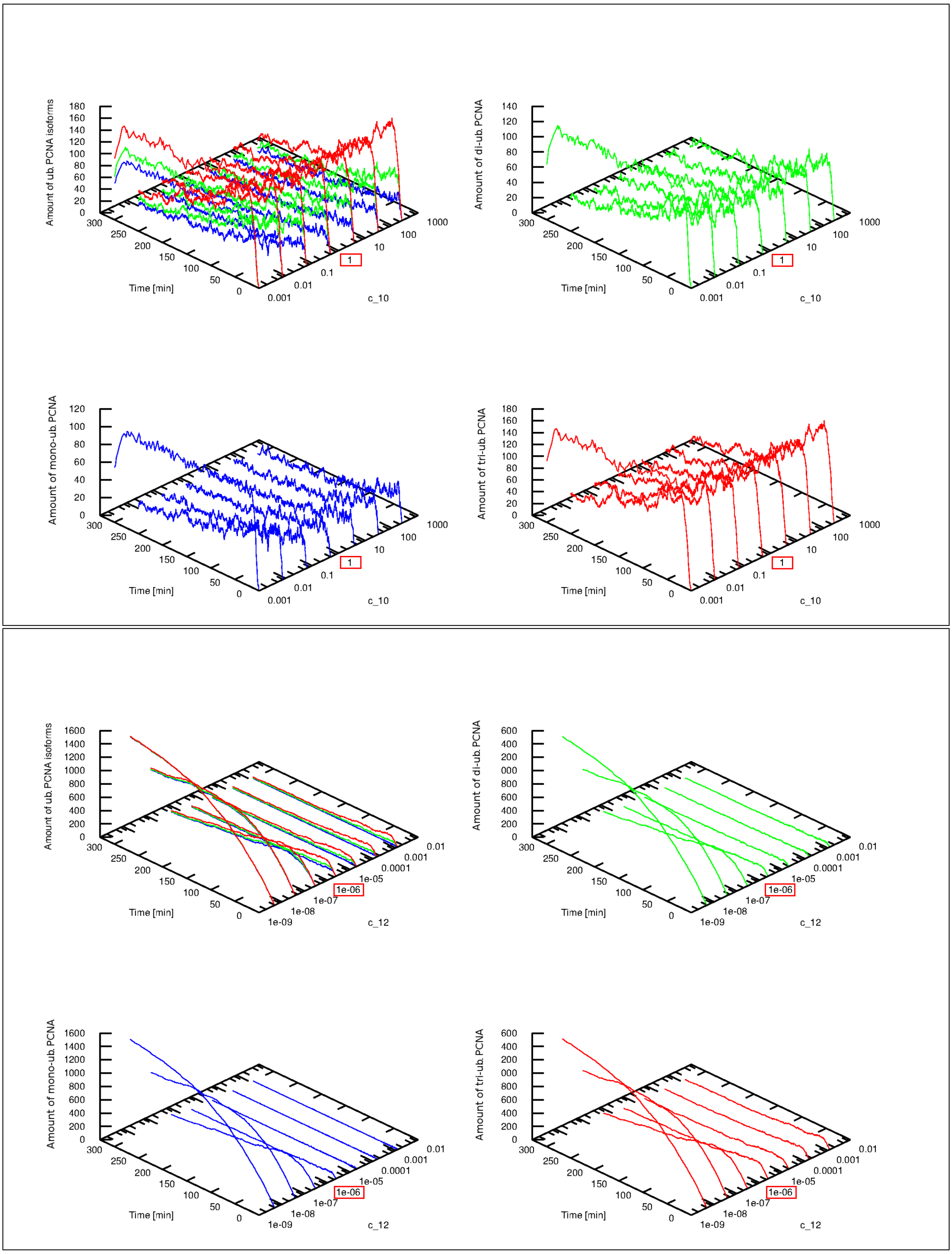

Supplement: Additional file 14 — Effects of the modulation of the dissociation of mono-ubiquitylated PCNA from Rad18 dimer (reaction constant c10) and of the association of Rad5 with mono-ubiquitylated PCNA (reaction constant c12). [file 1752-0509-7-24-S14.pdf]
