# Supplementary material for: In vivo and in silico analysis of PCNA ubiquitylation in the activation of the Post Replication Repair pathway in S. cerevisiae
Source: BMC Syst Biol. 2013 Mar 20;7:24. doi: 10.1186/1752-0509-7-24 (PMC3668150; doi:10.1186/1752-0509-7-24)

ADDITIONAL FILE 15

Effects of the modulation of the formation of di-ubiquitylated (reaction constant c_{16}) and of tri-ubiquitylated (reaction constant c_{22}) PCNA isoforms

The figure shows the simulated dynamics of the ubiquitylated PCNA isoforms resulting from two PSA, the first executed on the value of the reaction constant that modulates the formation of di-ubiquitylated PCNA isoform (top frame), and the second one executed on the reaction constant related to the formation of tri-ubiquitylated PCNA isoform (bottom frame). All simulations were executed assuming an UV dose equal to 10 J/m^2.

The two varied parameters and the corresponding sweep ranges are: (1) constant c_{16}, within the interval $[5 \times 10^{-5}, 50]$; (2) constant c_{22}, within the interval $[5 \times 10^{-6}, 5]$. The dynamics corresponding to the reference values (see Table 3) are marked with a red box.

For values of reaction constant c_{16} lower or equal to 1×10^{-3} we observe an accumulation of mono-ubiquitylated PCNA, since the rate of formation of di-ubiquitylated PCNA is very low. Conversely, for higher values of this constant, the resulting dynamics are comparable to the reference one.

At very low values of reaction constant c_{22} (equal or lower than 1×10^{-4}), the tri-ubiquitylated PCNA isoform accumulates in the system, pushing the amount of the other two isoforms around zero. By lowering constant c_{22}, the system reduces the production of the complex $PCNA_{\text{on}}{:}U{:}U{:}U$; however, the high signal of tri-ubiquitylated PCNA isoform is due to the accumulation of $Rad5{:}PCNA_{\text{on}}{:}U{:}U{:}U$, whose level, in turn, rises because this complex cannot undergo degradation. On the other hand, higher values of constant c_{22} result in a dynamics comparable to the reference one, probably for the combined interplay with reaction 25.

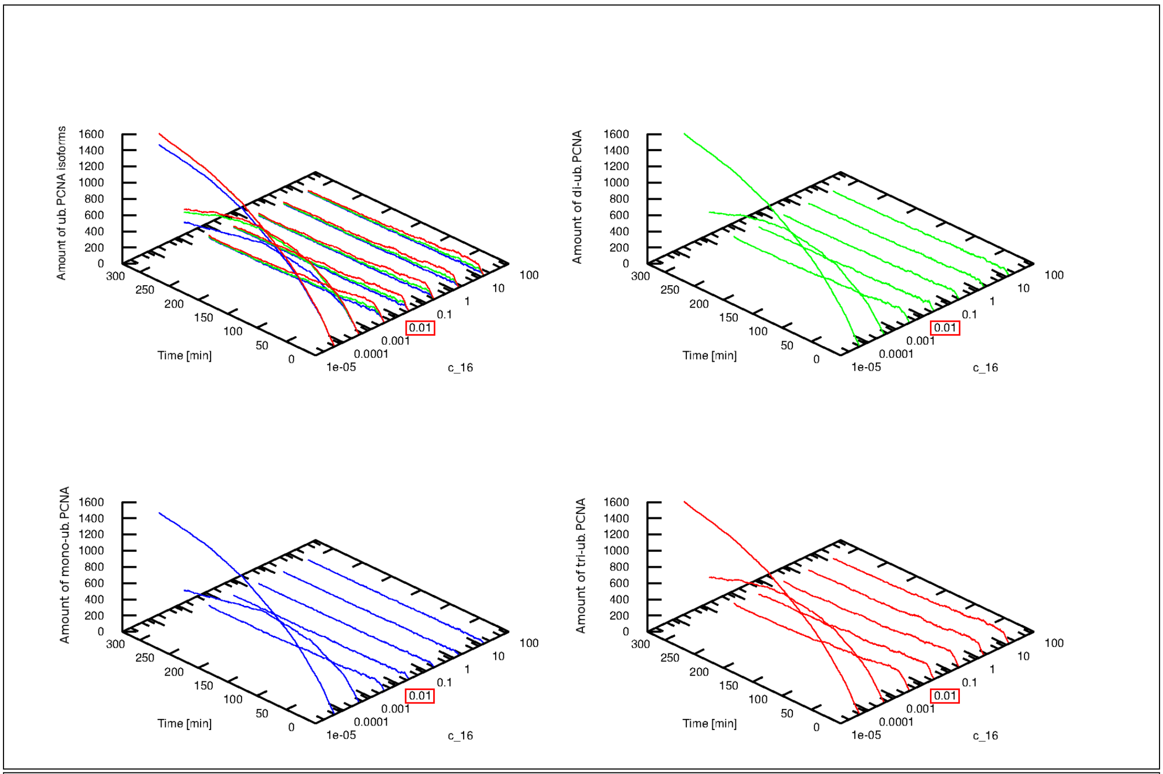

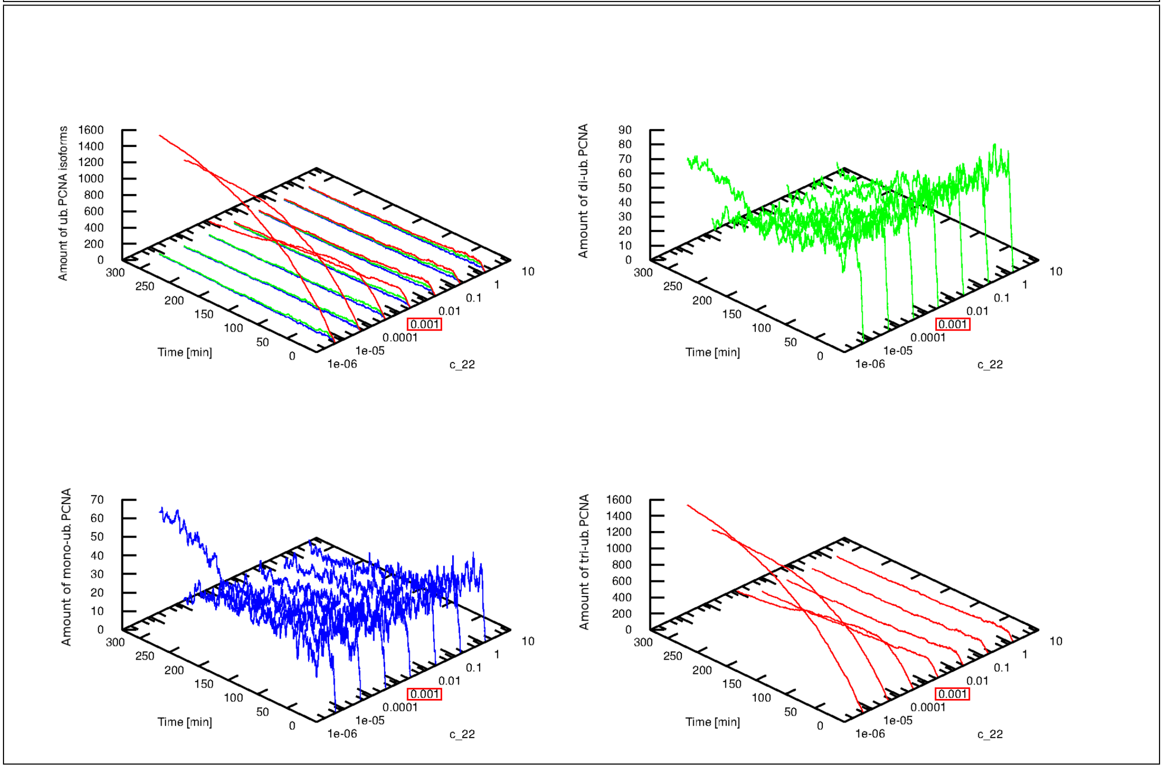

Supplement: Additional file 15 — Effects of the modulation of the formation of di-ubiquitylated (reaction constant c16) and of tri-ubiquitylated (reaction constant c22) PCNA isoforms. [file 1752-0509-7-24-S15.pdf]
