# Supplementary material for: In vivo and in silico analysis of PCNA ubiquitylation in the activation of the Post Replication Repair pathway in S. cerevisiae
Source: BMC Syst Biol. 2013 Mar 20;7:24. doi: 10.1186/1752-0509-7-24 (PMC3668150; doi:10.1186/1752-0509-7-24)
Supplement: Additional file 16 — FACS analysis of wild type andrad14δ background cells irradiated at 10 J/m2 UV dose. [file 1752-0509-7-24-S16.pdf]

ADDITIONAL FILE 16

FACS analysis of wild type and *rad14*Δ background cells irradiated at 10 J/m^2 UV dose

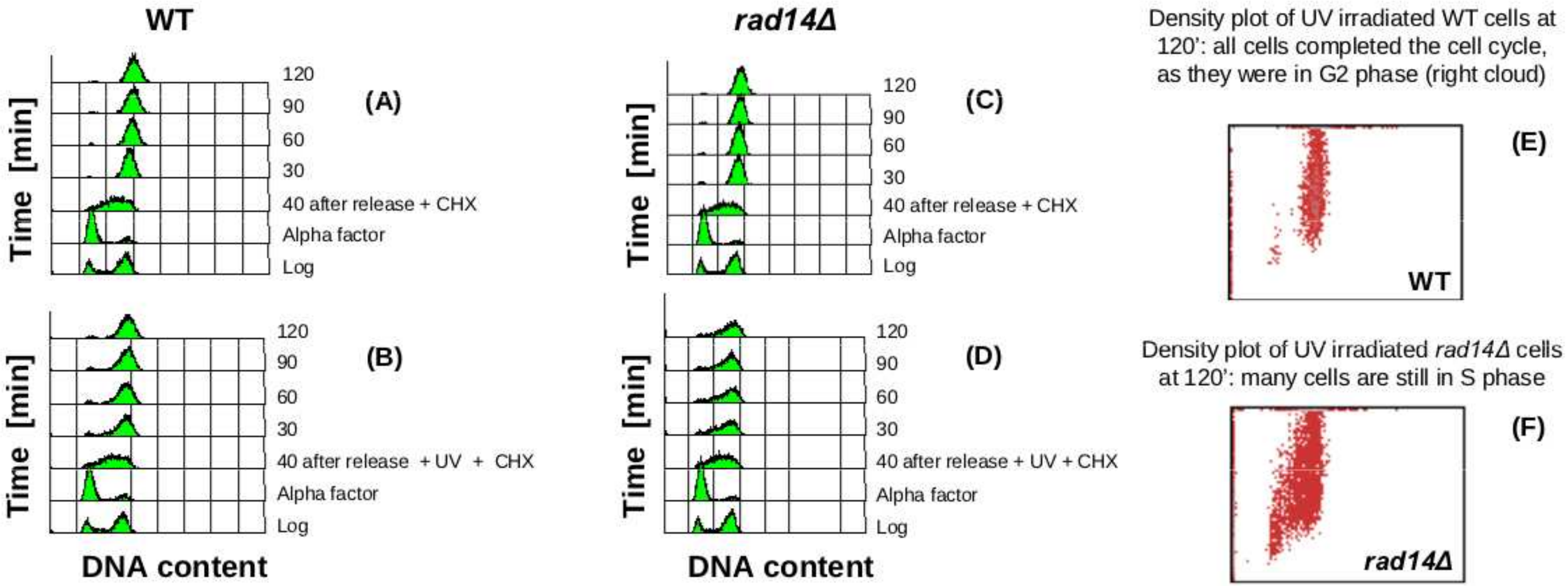

WT and *rad14*Δ cells were synchronized through 6 μg/ml of alpha factor. Cells were released in liquid medium till more than 80% of both cell populations were in S phase (about 30 minutes). Cells were then harvested and half of both populations were UV irradiated at 10 J/m^2 and released in liquid medium plus 10 μg/ml of CHX, while the other half was released in liquid medium plus 10 μg/ml of CHX without UV irradiation. Non irradiated cells of both populations completed the cell cycle within two hours (**A** and **C**) and UV irradiated WT cells completed the cell cycle with no difficulties (**B** and **E**). On the contrary, S phase UV irradiated *rad14*Δ cells had many troubles to complete the cell cycle and at two hours after UV irradiation many of them were still in S phase (**D** and **F**).
This experiment shows that UV irradiated *rad14*Δ cells in S phase show the same defect of UV irradiated *rad14*Δ cells in G1 phase [1], suggesting that NER must "clean" the genome from UV adducts also in S phase for the completion of DNA replication.

[1] Neecke H, Lucchini G, Longhese M: **Cell cycle progression in the presence of irreparable DNA damage is controlled by a Mec1- and Rad53-dependent checkpoint in budding yeast.** *EMBO J* 1999, 18(16):4485–97.
