# Supplementary material for: In vivo and in silico analysis of PCNA ubiquitylation in the activation of the Post Replication Repair pathway in S. cerevisiae
Source: BMC Syst Biol. 2013 Mar 20;7:24. doi: 10.1186/1752-0509-7-24 (PMC3668150; doi:10.1186/1752-0509-7-24)
Supplement: Additional file 17 — Deletion ofUBP10 andUBP15 do not prevent PCNA deubiquitylation at later time-points after low acute UV irradiation. [file 1752-0509-7-24-S17.pdf]

ADDITIONAL FILE 17

Deletion of *UBP10* and *UBP15* do not prevent PCNA deubiquitylation at later time-points after low acute UV irradiation

The figure shows the time-course measurement of mono-, di- and tri-ubiquitylated PCNA isoforms (top part, denoted by α-Ub) and of non modified PCNA (bottom part, denoted by α-His), sampled from 0 to 5 h after UV irradiation.

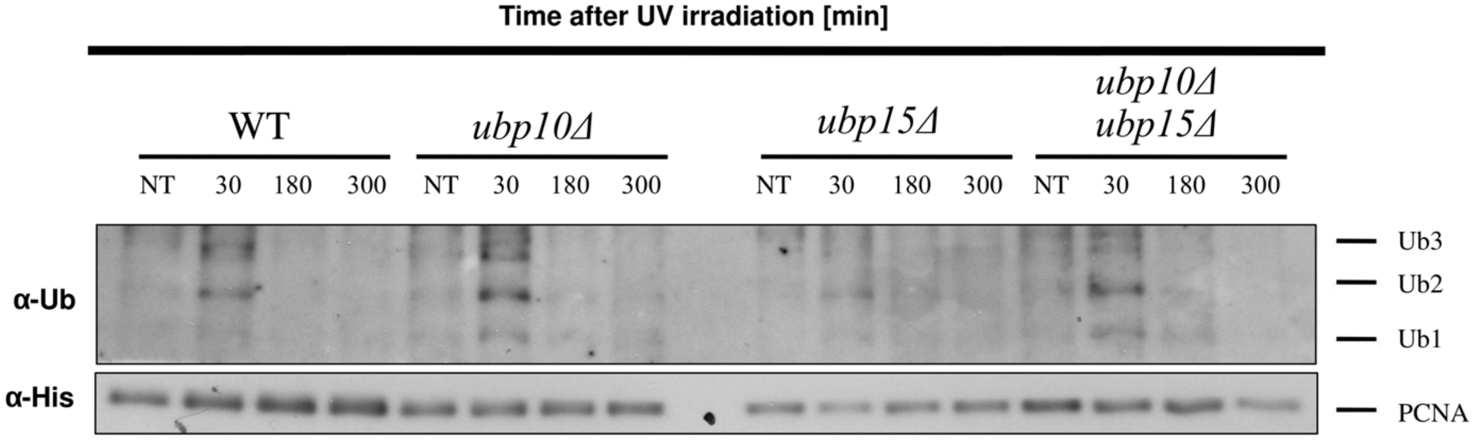

Wild type, single mutant and double mutant yeast cells were UV irradiated at 5 J/m^2, and collected at the indicated time-points. PCNA ubiquitylated isoforms were detected after denaturating pull-down, SDS-urea page and western blotting, as described in Section “Methods”.
